# Next-generation sequencing and bioinformatics capacity: findings from a multi-country survey to guide the genomics costing tool 2.0

**DOI:** 10.3389/fpubh.2026.1838184

**Published:** 2026-06-25

**Authors:** Ashley Bolding, Silvia Argimón, Toni Whistler, Beatrix Kele, Marco Marklewitz, Alexandr Jaguparov, Biran Musul, Anita Suresh, Swapna Uplekar, Joanna Salvi Le Garrec, Josefina Campos, Oluwatosin Wuraola Akande

**Affiliations:** 1 Global Health, Association of Public Health Laboratories, Bethesda, MD, United States; 2 WHO Hub for Pandemic and Epidemic Intelligence, World Health Organization, Berlin, Germany; 3 Virus Reference Department, United Kingdom Health Security Agency, London, United Kingdom; 4 Genomics & Sequencing, FIND, Geneva, Switzerland; 5 WHO Regional Office for Europe, World Health Organization, Copenhagen, Denmark; 6 WHO Country Office in Türkiye, World Health Organization, Ankara, Türkiye; 7 Genomics & Sequencing, FIND, Singapore, Singapore; 8 Department of Epidemic and Pandemic Management, World Health Organization, Geneva, Switzerland

**Keywords:** bioinformatics, computation biology, costing tool, genomic surveillance, laboratory budget, next-generation sequencing, public health surveillance, sustainability

## Abstract

Next-generation sequencing (NGS) and bioinformatics are critical to infectious disease surveillance, outbreak detection and response, and the research and development of medical countermeasures. Achieving sustainable genomic surveillance requires countries to develop costed national strategies that integrate financial planning and budgeting across sequencing and bioinformatics activities. The genomics costing tool (GCT) was initially developed to estimate the costs of SARS-CoV-2 sequencing and associated bioinformatics. In response to growing country demand, the tool has now been expanded to support a wider range of pathogens and laboratory settings (GCT 2.0). To inform the design of GCT 2.0, a cross-sectional online survey was disseminated between September 2024 and March 2025 to assess current global next-generation sequencing and bioinformatics capacity. Respondents were recruited via professional networks, mailing lists, and partner organizations. The questionnaire captured laboratory demographics, instrumentation, reagents, throughput, data management, bioinformatics/analytical tools, and funding sources. Of the 149 respondents, 120 responses from 52 countries across all six WHO regions were included in the analysis, after excluding incomplete submissions. The median number of sequencing instruments per respondent was 3, with Illumina and Oxford Nanopore Technologies being the most predominant platforms, reported by 89.6 and 68.8% of the respondents, respectively. The median annual throughput reported was 1,940 samples in high-income countries, 850 in upper-middle-income countries, 1,205 in lower-middle-income countries, and 950 in low-income countries. Only 57.7% of respondents stored data in multiple locations, and 32.5% lacked any data backup. Funding sources varied: 54.4% relied on multiple streams, while 14.9% depended solely on government budgets, and many laboratories relied on emergency or project-based support. Global NGS and bioinformatics capacity continues to expand, yet substantial geographical and operational disparities persist. Beyond instrument availability, laboratories face constraints related to throughput, data storage, analysis capacity, and sustainable financing. Informed by these findings, GCT 2.0 incorporates expanded pathogen coverage, flexible throughput scenarios, support for multiple sequencing platforms, and detailed costing of data storage and bioinformatics workflows. By integrating these considerations, the tool aims to strengthen laboratories’ capacity to plan, manage, and sustain genomic surveillance over the long term.

## 1 Introduction

The rapid advancement of next-generation sequencing (NGS) technologies has revolutionized the fields of genomics, transcriptomics, and metagenomics, offering unprecedented opportunities for understanding biological systems (1). These technologies have transformed public health, enabling real-time pathogen detection, characterization and monitoring, development of medical countermeasures, as well as research to inform decision-making for public health priorities (2).

Translating these advances into effective and sustainable impact requires equitable access to sequencing platforms, reagents and consumables, workforce expertise, and robust bioinformatic infrastructure to manage and interpret the vast amounts of generated data. Though numerous countries, donors and institutions have made substantial investments in expanding sequencing and bioinformatics capabilities, there are significant disparities in access to these resources across regions and this could constrain the generation, analysis and use of genomic data (3).

Achieving sustainable genomic surveillance requires countries to develop costed national strategies that integrate financial planning and budgeting across sequencing and bioinformatics activities (2). Realistically costing those strategies, in turn, requires accurate, current data on what infrastructure and workflows laboratories are using. Previous assessments include gaps in pathogen specific data publications, such as SARS-CoV-2 data output on GISAID and GenBank (4, 5), regional in scope (5, 6), or focused narrowly on sequencing capacity without capturing the bioinformatics workflows and resource requirements that determine whether raw sequence data can be translated into actionable public health data.

The genomics costing tool (GCT) is a freely accessible, excel-based tool designed to support laboratories, ministries of health, donors, and decision-makers estimate the full costs of genomic sequencing and bioinformatics (7, 8). The first edition of the GCT was developed specifically to support the costing of severe acute respiratory syndrome coronavirus 2 (SARS-CoV-2) sequencing and bioinformatics activities (7, 8). It offered only fixed throughput increments, supported only Illumina and ONT platforms, and did not incorporate customizable platforms, reagent inputs, detailed bioinformatics infrastructure costing, or average-based run loading calculations, all of which are critical for accurately costing genomic surveillance programmes across diverse laboratory settings. Following its release, countries and partners requested an expanded version (GCT 2.0^1^) that could support cost estimation for a broader range of pathogens, sequencing technologies, and applications (9). Developing a tool that is globally relevant required an up-to-date understanding of current NGS and bioinformatics infrastructure and practices to ensure the tool is practical, adaptable and of great value to laboratories globally. To fill this gap, we conducted a multilingual global survey to quantify global, cross-sectoral operational NGS and bioinformatics capacity and to parameterize priorities for GCT 2.0. This manuscript presents the findings of this assessment. Beyond informing the development of GCT2.0, these data address a broader need to evaluate the current state of NGS and bioinformatic capacities, to identify critical gaps and challenges and to support the design of sustainable capacity strengthening strategies.

## 2 Methods

### 2.1 Survey design

The global cross-sectional online survey, conducted from 1 September 2024 to 21 March 2025, was designed to assess NGS and bioinformatics capacities across institutions covering diverse geographic, economic, and scientific contexts. The survey adhered to the Strengthening the Reporting of Observational Studies in Epidemiology (STROBE) guidelines for observational studies (10).

### 2.2 Study setting and dissemination of survey tool

Respondents were identified through professional networks (e.g., LinkedIn and professional forums), mailing lists, and partnerships with relevant organizations [the Association of Public Health Laboratories (APHL), FIND, the Global Fund to Fight AIDS, Tuberculosis and Malaria (TGF), the UK Health Security Agency (UKHSA), and the World Health Organization (WHO)]. Survey distribution was conducted through these global institutions using convenience and snowball sampling to ensure outreach to diverse stakeholders and geographies.

### 2.3 Participants and ethical considerations

Eligible participants included professionals involved in NGS workflows for public health, such as laboratory scientists, bioinformaticians, laboratory managers, and public health officials. The inclusion criteria required participants to have direct experience with NGS workflows, bioinformatics analysis, or the decision-making processes related to genomic surveillance. All participants provided informed consent electronically before accessing the survey. The purpose of the study, the voluntary nature of participation, and confidentiality measures were explained to the participants. Participants could withdraw at any time without penalty. No personally identifiable information was collected.

### 2.4 Survey instrument and data collection methods

The survey’s focus included basic laboratory demographics, pathogens sequenced, annual throughput, funding mechanisms, instrumentation, reagents and consumables used for extraction, library preparation, and sequencing, as well as analytical tools and data management practices.

The survey (Supplementary File 1) was designed, and responses were collected using SurveyMonkey, which was selected for its user-friendliness, question logic functionality, data security features, and ability to export data for analysis (11). The questionnaire was configured using skip logic to ensure respondents only answered follow-up questions relevant to their selected responses. The questions were a mix of closed- and open-ended, capturing quantitative and qualitative data.

The questionnaire was pretested and validated by the GCT core technical team to refine its clarity, relevance, and ease of understanding. Feedback from this process informed revisions to ensure that questions were clear and aligned with study objectives. The survey was developed in English and translated by fluent technical experts into French, Spanish, and Russian to facilitate participation from non-English-speaking regions (Supplementary Files 2–4).

A survey solicitation was created (Supplementary File 5) and shared via social networks, email, and mailing lists. The survey window closed on March 21, 2025.

### 2.5 Bias

To minimize selection bias, all questions were neutrally worded, and participants were informed of the confidentiality of their survey responses. Reminders were sent throughout the 7-month survey period to minimize non-response bias.

### 2.6 Data cleaning

Data cleaning involved a structured process to ensure data quality and integrity. The dataset was examined for completeness, structure, and any apparent anomalies. A threshold for acceptable completion was established, defined as completing the survey beyond the demographic questions. Responses with solely demographic information were excluded from the analysis. Open-ended responses were reviewed for translation accuracy, typos, and irrelevant content. Branching logic was verified to ensure consistency in responses. For example, if participants indicated “No” to having a particular instrument, related follow-up questions were checked; inconsistent responses were either flagged for discussion or removed. Sequencing and library prep kit compatibility with reported instrument(s) was double-checked. If there was no concordance, the response was removed (*n* = 1). Identifiable information, if inadvertently provided, was removed to maintain anonymity.

A record of all data-cleaning actions was maintained to document modifications and ensure transparency in the cleaning process.

### 2.7 Data analysis

Data manipulation, analysis, and visualization were conducted with R version 4.5.0, RStudio version 2025.05.1 + 513, the tidyverse software packages v2.0.0, the ggVennDiagram package v1.5.4, and the whomapper package v0.1.1. Descriptive statistics (e.g., frequencies, percentages, and measures of central tendency) were used to summarize the respondent characteristics and survey responses. Missing data (NA) were ignored for descriptive summaries and visualization on an individual (question-by-question) basis.

For open-ended questions, thematic analysis was employed to identify common themes and insights regarding NGS and bioinformatics capacities.

### 2.8 Ethical approval

A waiver was granted by the WHO Ethics Review Committee (Project ID: ERC.0003954) to publish the findings from this activity.

## 3 Results

### 3.1 Total respondents

Out of the 149 individuals who accessed the survey, 120 met inclusion criteria and completed all required sections for analysis (93% completion rate). Exclusions included respondents with only demographic data (*n* = 11), respondents intending to establish NGS capacity but lacking instrumentation (*n* = 9), duplicate entries (*n* = 3), respondents using only Sanger sequencing but with no NGS capability (*n* = 2), respondents outsourcing sequencing to commercial entities (*n* = 2), and respondents with nonconforming responses across survey items, which limited interpretability of the data (*n* = 2). The median completion time for the survey was 17 min (interquartile range (IQR) = 8–32 min).

### 3.2 Respondent characteristics

**Figure 1:**
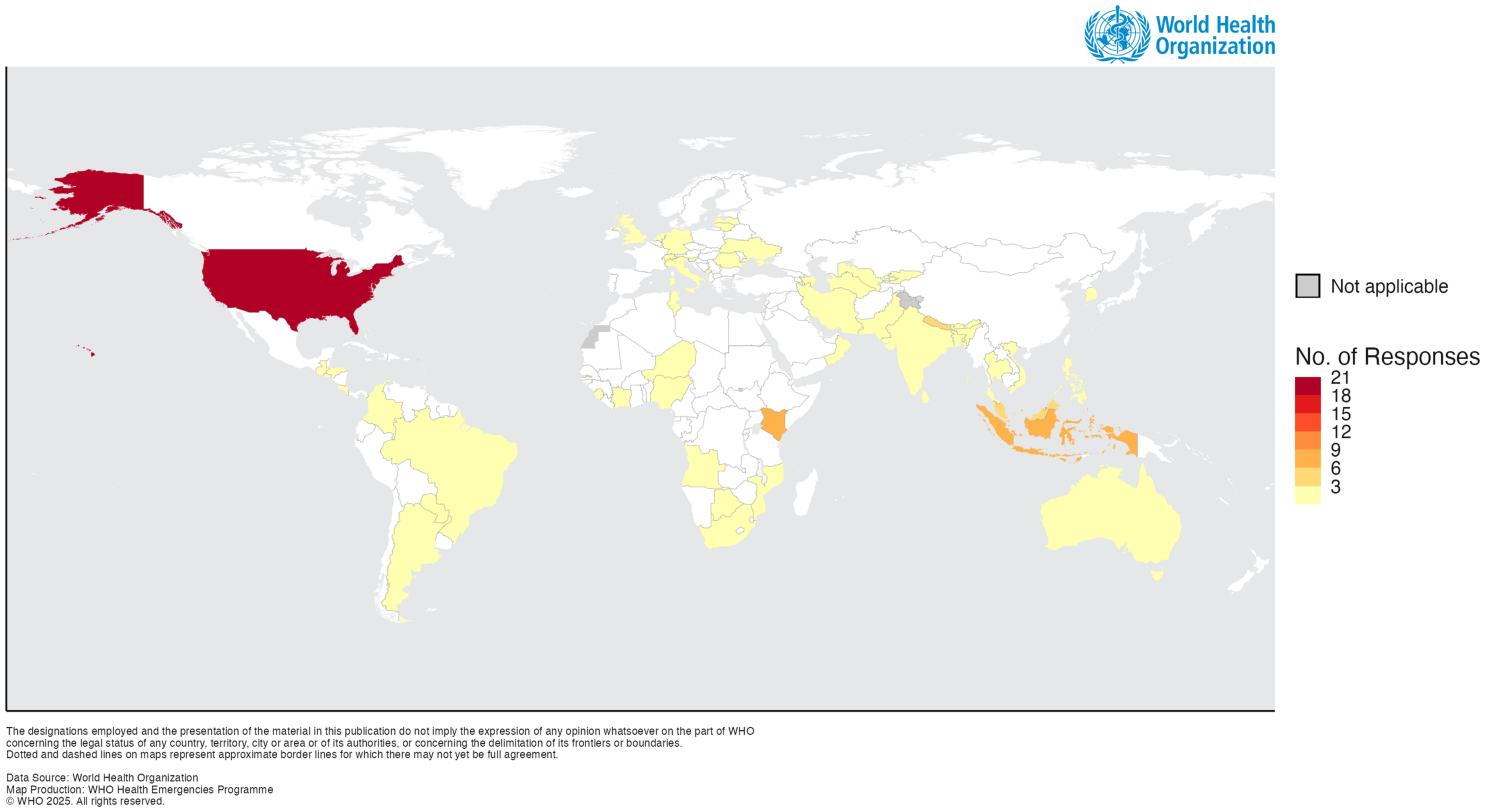
Geographical distribution of 120 participating laboratories included in this study.

The respondents represented institutions across 52 countries (Figure 1), spanning all six WHO regions: 17.5% (*n* = 21) from the African Region (AFR), 27.5% (*n* = 33) from the Region of the Americas (AMR), 3.3% (*n* = 4) from the Eastern Mediterranean Region (EMR), 19.2% (*n* = 23) from the European Region (EUR), 20.8% (*n* = 25) from the South-East Asian Region (SEAR), and 11.7% (*n* = 14) from the Western Pacific Region (WPR, Figure 1).

**Figure 2:**
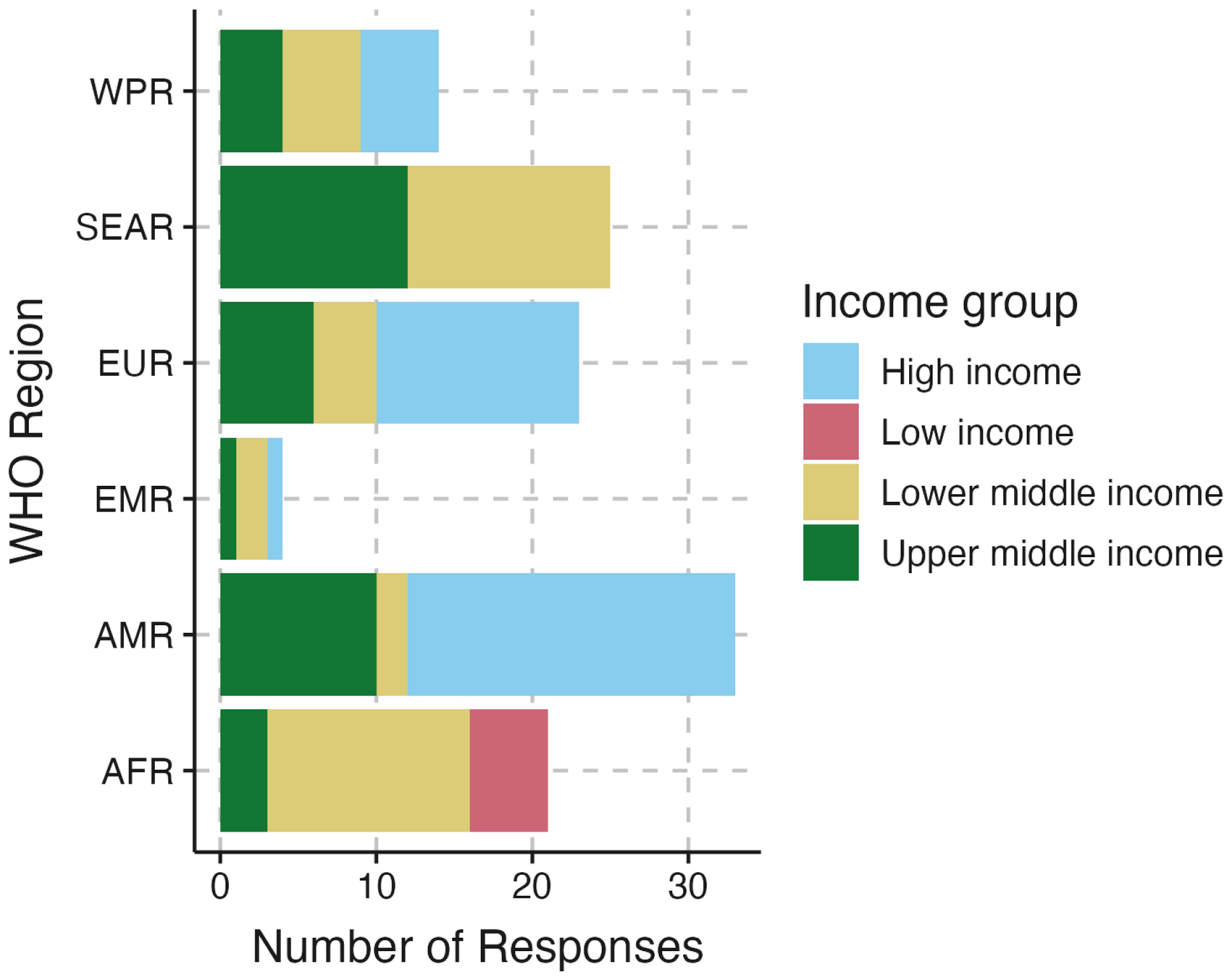
Distribution of responses by WHO Region and country income category.

Responses were received across all country income levels as designated by the World Bank for 2024–2025 (12): Respondents from high-income countries (HICs) accounted for 33.3% of the responses (*n* = 40), followed by respondents in lower-middle income countries (LMICs, 32.5%, *n* = 39), upper-middle-income countries (UMICs, 30.0%, *n* = 36) and low-income countries (LICs, 4.2%, *n* = 5). Respondents in LICs were only from the AFR and accounted for 23.8% (5/21) of the responses from this region (Figure 2).

Most responses were from national public health institutions (60.0%, *n* = 72), followed by subnational and research laboratories (10.0%, *n* = 12).

### 3.3 Sequencing instrumentation

Overall, 96 of 120 respondents (80%) indicated at least one sequencing instrument model. Illumina and Oxford Nanopore Technologies (ONT) instrumentation platforms were the most reported. Illumina instruments were reported by 86 respondents (89.6%) across 45 countries, with 38 respondents (39.6%) reporting the presence of two or more Illumina instrument models. The MiSeq was the most widely reported Illumina model (*n* = 69, 71.9%), followed by NextSeq (500/550/1000/2000) (*n* = 42, 43.8%), iSeq (*n* = 27, 28.1%), and MiniSeq (*n* = 11, 11.5%) (Table 1).

**Table 1:** Distribution of sequencing instrument models by manufacturer.

Manufacturer	Instrument model	No. of responses (*n* = 96)	% Respondents
Illumina	iSeq	27	28.1
	MiniSeq	11	11.5
	MiSeq	69	71.9
	NextSeq^a^	42	43.8
	Other^b^	9	9.4
Oxford Nanopore Technologies (ONT)	MinION^c^	59	61.5
	GridION	27	28.1
	PromethION^d^	14	14.6
Thermo Fisher^e^	IonChef	7	5.8
	Ion OneTouch	3	3.1
	GeneStudio	6	6.2
	Genexus	3	3.1
MGI Tech	DNBSEQ G50	2	2.1
	DNBSEQ G400	1	1.0
Pacific Biosciences (PacBio)^f^		3	3.1
ABI	Sanger sequencer	38	30.6
Clear Labs	Clear Dx	1	0.8

Each instrument model was computed individually when the same respondent reported multiple models.

a NextSeq includes the 500 (*n* = 5), 550 (*n* = 12) and 1,000/2,000 (*n* = 25) instruments.

b Other includes the MiSeq i100 (*n* = 2) and NovaSeq 6000 (*n* = 7).

c MinION instruments were the Mk1B (*n* = 25), Mk1C (*n* = 32), and Mk1D (*n* = 2).

d PromethION includes both the P2 and P2 Solo instruments.

e Different configurations of an instrument were counted together.

f No model information was provided for PacBio.

ONT instruments were reported by 66 respondents (68.8%) across 41 countries, with multiple ONT instrument models reported by 23 respondents. MinION devices (primarily Mk1C and Mk1B) were the most reported ONT instruments (*n* = 59, 61.5%), followed by GridION (*n* = 27, 28.1%) and PromethION (*n* = 14, 14.6%). Fifty-six respondents (58.3%) across 36 countries reported the availability of both Illumina and ONT instruments.

Thermo Fisher and MGI instruments were less common, reported by 10 (10.4%) and 3 (3.1%) respondents, respectively. PacBio instruments were reported by 3 (3.1%) respondents, and only one respondent (1.0%) reported using Clear Dx (Clear Labs). In addition to NGS instrumentation, 37 respondents (38.5%) reported having an ABI Genetic Analyzer (for Sanger sequencing) or access to one (*n* = 1).

**Figure 3:**
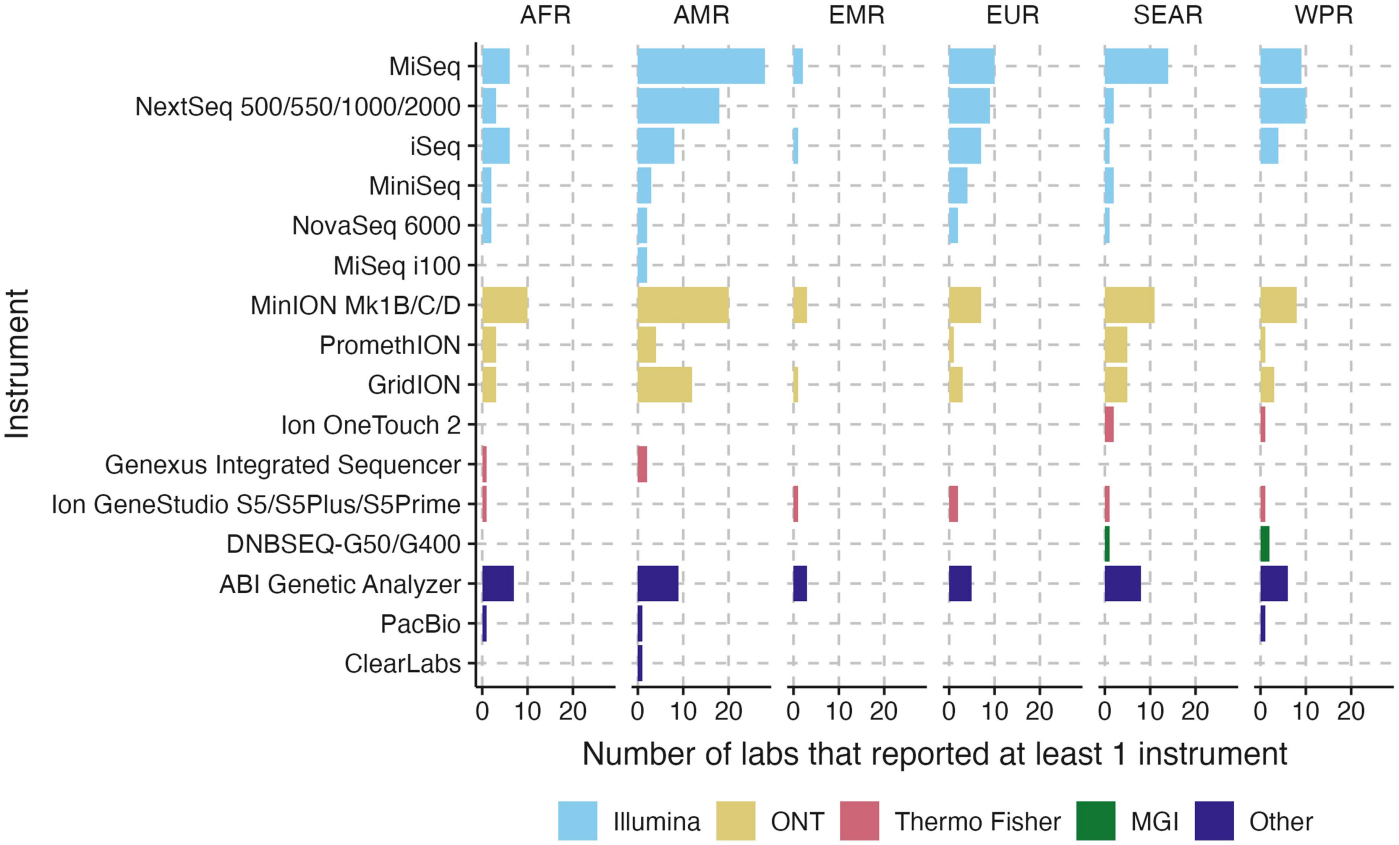
Sequencing instruments models reported in laboratories in six WHO regions. Similar models were grouped. Colors indicate different manufacturers.

Availability of Illumina technology varied by income level: 60.0% (3/5) of respondents from LICs, 62.5% (25/39) from LMICs, 61.1% (22/36) from UMICs, and 90.0% (36/40) from HICs reported using Illumina instruments. In contrast, ONT presence was more similar across income levels: 52.5% (21/40) in HICs, 58.3% (21/36) in UMICs, 53.8% (21/39) in LMICs, and 60.0% (3/5) in LICs. Illumina, ONT, Thermo Fisher, and ABI instruments (for Sanger sequencing) were represented across all six WHO regions. However, MGI instruments were reported only in the Southeast Asia and Western Pacific regions, and PacBio instruments were reported only in the Africa, Americas, and Western Pacific regions (Figure 3).

**Figure 4:**
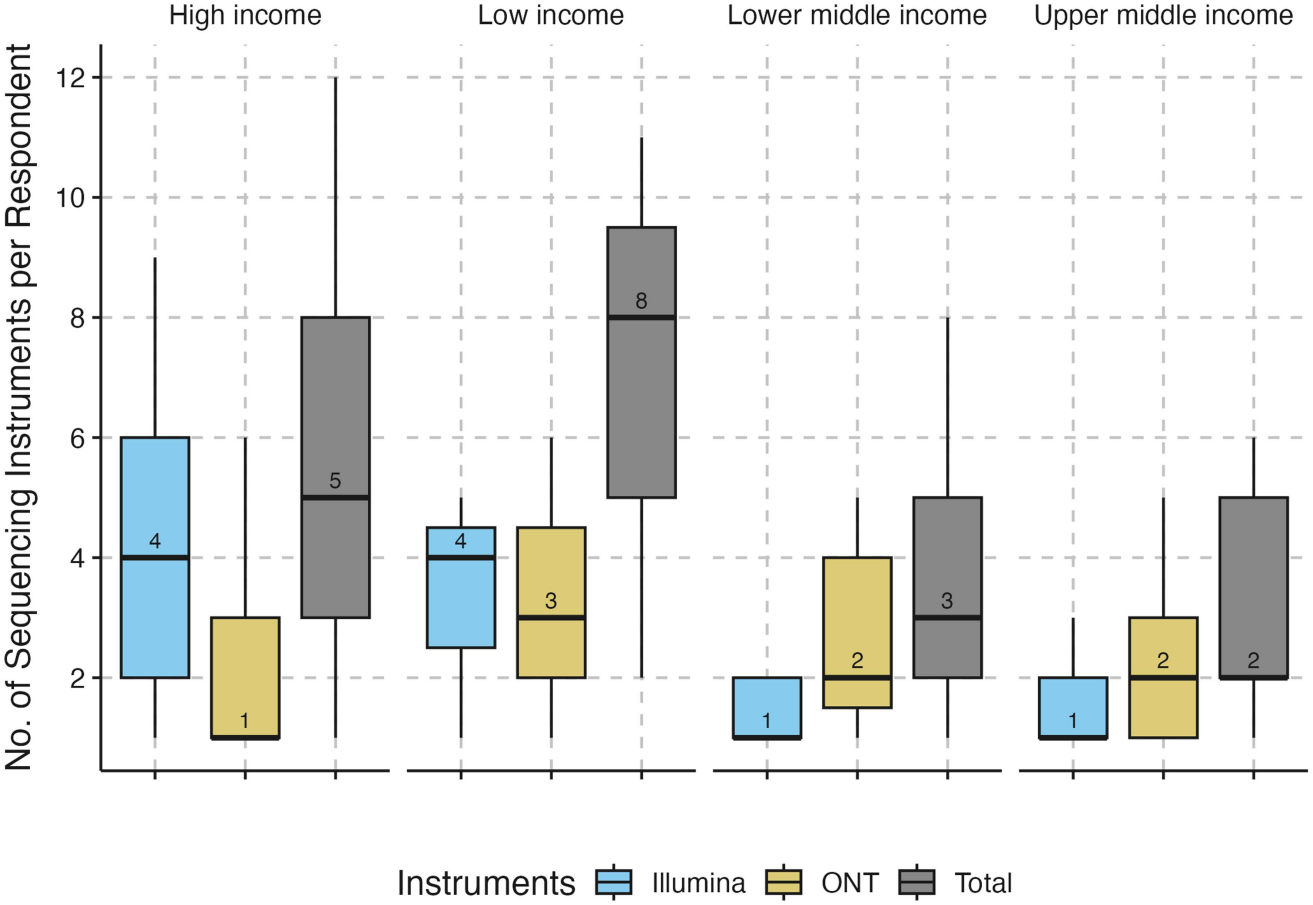
Distribution of the number of sequencing instruments per respondent by country income level. The distributions of Illumina, ONT, and the total number of instruments (which includes Illumina, ONT, MGI and Thermo Fisher platforms) are shown. The medians for each group are represented by the horizontal lines and annotated with their corresponding values. The boxes span the first to third quartiles. The upper and lower whiskers extend to the largest or smallest value no further than 1.5 times the inter-quartile range. Data beyond the whiskers were considered outliers and were removed from the plot but included in the summary statistics.

Respondents also provided information on the total number of available NGS instruments from four manufacturers (Illumina, ONT, ThermoFisher, and MGI). The median number of NGS instruments per respondent was 3 (IQR 2–6). Respondents in LICs reported the highest median number of instruments (8, IQR 5–9.5), followed by HICs with a median of 5 (IQR 3–8) (Figure 4).

### 3.4 Sequencing loading capacity by manufacturer and income level

Optimization of sequencer loading capacity varied by manufacturer and income level (Table 2). Nearly half of Illumina users responding to the question about loading capacity reported frequently loading to full capacity (48.6%, 34/70), compared with 28.8% (17/59) of ONT users who responded to the same question. Optimization was generally highest in HICs and UMICs, while partial or underloading was more frequent among LMICs. The number of responses from LICs (*n* = 3) was insufficient to allow meaningful statistical analysis. Of the 10 (*n* = 10) responses regarding Thermo Fisher loading capacity, two (20%) indicated that loading capacity was always optimized, four (40%) reported it was sometimes optimized, and four (40%) stated that it was not optimized. Of the three (*n* = 3) responses regarding MGI loading capacity, two (66.7%) indicated that it was always optimized, while 1 (33.3%) reported that it was not optimized.

**Table 2:** Sequencing loading capacity by manufacturer and income level for Illumina and ONT instruments.

Country income development level	Optimization of sequencer loading capacity							
	Illumina (*n* = 70 respondents)				ONT (*n* = 59 respondents)			
	No	Some-times	Yes	Non-response	No	Some-times	Yes	Non-response
High-income	1	13	13	13	4	10	4	22
Upper-middle income	2	7	12	15	4	9	7	16
Lower-middle income	5	7	7	21	1	12	5	22
Low-income	0	1	2	2	0	2	1	2
Total no. responses	8	28	34		9	33	17	
% Total	11.4	40.0	48.6		15.3	55.9	28.8	

### 3.5 Library preparation and sequencing kits

Seventy-five (*n* = 75) respondents provided information regarding library preparation and sequencing kits. The top three library preparation kits reported for Illumina instruments were DNA Prep (*n* = 50, 66.7%), NexteraXT (*n* = 29, 38.7%), and COVIDSeq (*n* = 28, 37.3%). For sequencing kits, the most reported were MiSeq v3 (600c) (*n* = 30, 42.9%), MiSeq v2 (300c) (*n* = 29, 41.4%), and MiSeq v2 500c (*n* = 23, 32.9%). The most reported ONT library preparation and sequencing kits were the Ligation Sequencing Kit (*n* = 33, 44%), the Rapid Barcoding Kit (*n* = 30, 40%), and the Midnight RT PCR Expansion Kit (*n* = 18, 24%). Although the number of Thermo Fisher and MGI library kits reported was low (*n* = 14 and *n* = 5, respectively), several kits, including the Ion AmpliSeq™ Library Kit 2.0, Ion Xpress™ Plus Fragment Library Kit, Ion Xpress™ Barcode Adapters 1–16 Kit, and the MGIEasy Universal DNA Library Prep Set, were each reported by more than one respondent (Supplementary File 6).

### 3.6 Sequencing throughput

Of the 98 respondents who reported annual throughput, 54 (55.5%) sequenced fewer than 1,000 samples per year, 22 (22.4%) sequenced between 1,001 and 4,000 samples per year, and 12 (12.2%) exceeded 4,000 samples per year (Table 3). All four (100%, 4/4) respondents from LICs, 90.3% (28/31) from LMICs, and 84.6% (22/26) from UMICs reported annual throughputs below 2,000. In contrast, only about half of the respondents from HICs (20/37, 54.1%) reported annual throughputs below 2,000.

**Table 3:** Current annual specimen sequencing throughput reported by 98 respondents.

Annual throughput	Low income	Lower middle income	Upper middle income	High income	Total	% (*n* = 98)
0—looking to establish a sequencing laboratory	1	5	4	0	10	10.2
1–100	0	5	6	5	16	16.3
101–600	1	11	9	3	24	24.5
601–1,000	1	5	2	6	14	14.3
1,001–2,000	1	2	1	6	10	10.2
2001–3,000	0	1	1	4	6	6.1
3,001–4,000	0	0	1	5	6	6.1
4,001	0	2	2	8	12	12.2
Total	4	31	26	37	98	

This was also reflected in the total number of samples reported across different pathogens (see Section 3.7): HICs reported the highest median number of samples per year (1940, IQR 1121.3–3663.8). LMICs reported a median of 1,205 samples per year (IQR 410.3–4752.5), UMICs reported a median of 850 samples/year, and LICs reported a median of 950 samples per year (IQR 490–1,350).

### 3.7 Pathogens sequenced

Overall, respondents reported performing more viral genome sequencing (annual median number of samples 900, IQR 305–2,000) than bacterial genome sequencing (annual median number of samples 675, IQR 262.5–1706.2). Most respondents (80.9%) reported sequencing SARS-CoV-2 specimens (Table 4), with 3 (2.5%) indicating it was the sole pathogen they surveilled and sequenced. Other common targets included influenza (sequenced by 67.3% of respondents), enteric bacteria (46.4%), *Mycobacterium tuberculosis* (MTB, 34.5%), and HIV (14.5%) (Table 4). Ten respondents indicated a focus exclusively on a single pathogen or pathogen group, such as arboviruses, MTB drug resistance, enteric bacteria, poliovirus, or *Burkholderia pseudomallei.*

**Table 4:** Summarized responses to genomic surveillance of pathogens.

Pathogens	No. of respondents	%(*n* = 110)
Severe acute respiratory syndrome coronavirus 2	89	80.9
Influenza virus	74	67.3
Other respiratory viruses	40	36.4
Enteric bacteria	51	46.4
Hospital-acquired infection (HAI) pathogens	39	35.5
HIV genotyping	16	14.5
HIV drug resistance	27	24.5
Arboviruses	36	32.7
*Mycobacterium tuberculosis* (MTB)	38	34.5
MTB drug resistance	40	36.4
Other	35	31.8

Percentages are calculated over the total number of responses to the question (*n* = 110).

**Figure 5:**
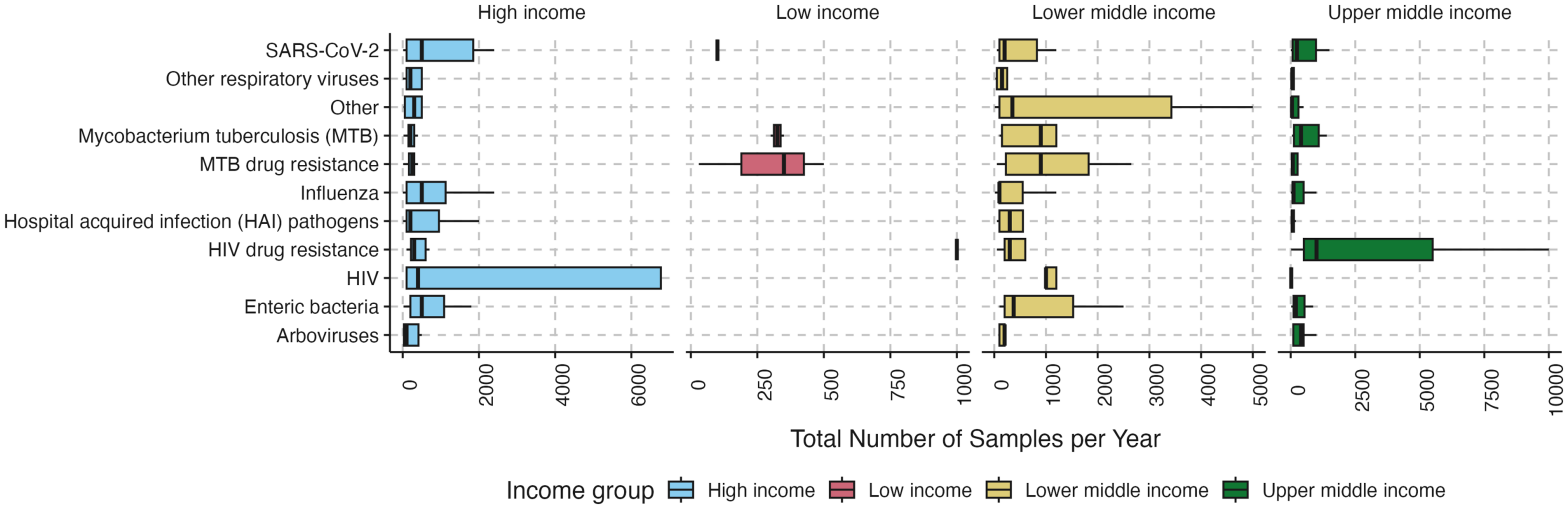
Total number of samples per year for each pathogen stratified by country income. The x-axis scales differ across different stratified groupings.

Within all income groups, sequencing throughput varied markedly by pathogen, but LICs showed sparser data (Figure 5). SARS-CoV-2 showed higher median throughputs than most other pathogens across all income groups except for LICs (Supplementary File 6). The annual throughputs of HIV and/or HIV drug resistance were also generally high across all income groups, but the presence of large upper tails in HICs and UMICs likely reflects that the sequencing output is likely concentrated in smaller number of laboratories. Higher median sequencing throughput was observed for MTB and/or MTB drug resistance in LICs, LMICs, and UMICs compared with HICs.

**Figure 6:**
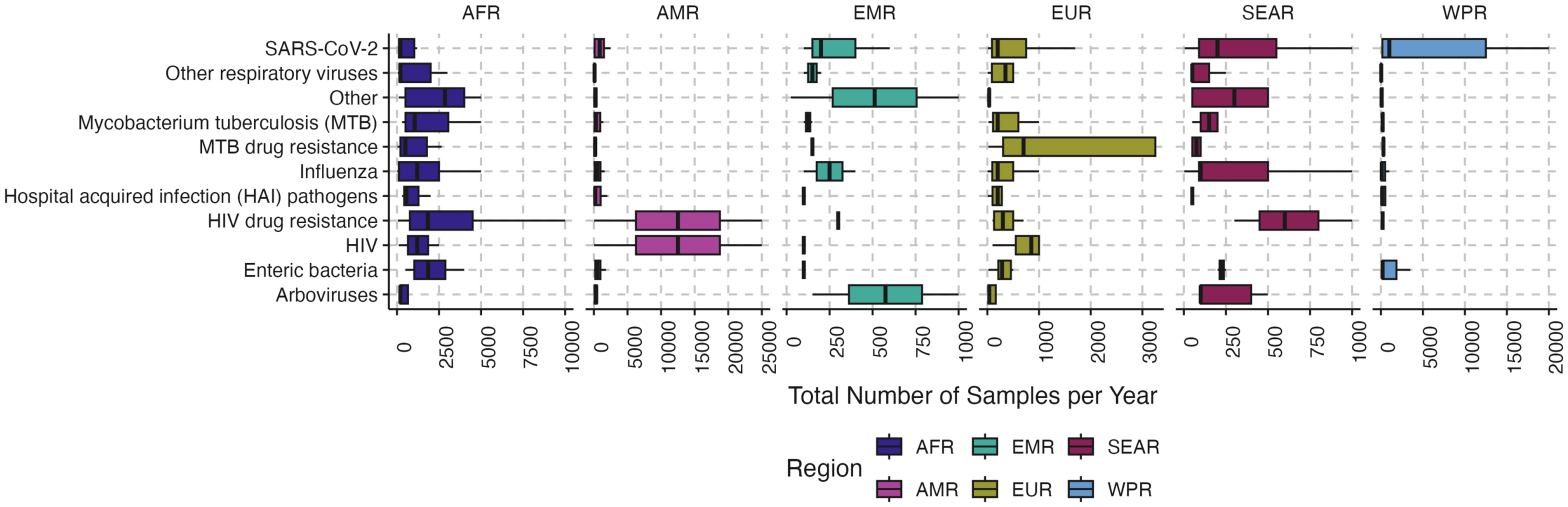
Total number of samples per year for each pathogen stratified by WHO region. The *x*-axis scales differ across different stratified groupings.

Within WHO regions, sequencing throughput also varied by pathogen, with EMR showing sparser data (Figure 6). SARS-CoV-2 showed higher median throughputs than all other pathogens in WPR (median 1,000) and AMR (median 860), the highest of all pathogens except HIV. The large upper tails observed in the SARS-CoV-2 throughput distribution across all regions (caused by high means and maximum values) likely reflect the fact that sequencing output is concentrated in a few laboratories. HIV and/or HIV drug resistance showed regional predominance in AMR, AFR and SEAR, with higher medians compared to most other pathogens (Supplementary File 6). Similarly, MTB and MTB drug-resistance showed regional predominance in EUR and WPR. Arboviruses generally showed low median throughput values (= < 350) across regions except for EMR (median 575), and were not reported by any respondent from WPR.

### 3.8 Data management and analysis

Data storage and backup practices varied widely. Of the 71 respondents who provided information on data storage, 30 (42.3%) reported storing sequence data in a single location, while 41 (57.7%) stored data in multiple locations. Of the 77 respondents who provided information on backup practices, 25 (32.5%) reported not using any form of data backup. Among the 52 (67.5%) respondents who backed up their data, 8 (15.4%) did not specify a location, 33 (63.5%) relied on a single location, and 17 (32.7%) used multiple locations. The data storage and backup locations varied among respondents, with external hard drives being the most used frequent solution for primary storage (38/71, 53.5%) and backup (26/52, 50.0%) (Table 5).

**Table 5:** Location of sequence data storage and backup.

Location	Data storage (*n* = 71)	Backup (*n* = 52)
File server	31	19
External hard drive	38	26
Computer hard disk	27	9
Cloud storage	28	14
Other	4	2

The totals represent the 71 participants who indicated at least one location for data storage, and the 52 participants who responded “yes” to data backup.

Based on responses from 76 participants regarding internet speed, 52 respondents (68.4%) representing all income levels reported download and upload speeds of more than 10 Mbps. In contrast, five respondents from LMICs and two from UMICs reported upload and download speeds below 10 Mbps.

Among the 79 respondents who provided information on computer workstations, 71 (89.9%) reported a dedicated workstation for analysis. Similarly, 70 respondents reported using at least one of the 20 analytical tools included in the survey or a self-reported tool (Table 6), with a median of 5 tools per respondent (IQR 4–7.75). Popular platform-independent tools used by at least 30% of the respondents included Nextstrain/Nextclade (*n* = 43), GISAID (*n* = 42), MEGA (*n* = 24), and Terra.bio (*n* = 22). Platform-specific tools were also commonly used, with MinKNOW reported by 37 respondents (53.6%), BaseSpace by 35 respondents (50.7%), and EPI2ME by 28 respondents (40.6%). Furthermore, of the 54 respondents, only 14 (25.9%) reported using a Laboratory Information Management System (LIMS) to link sequences to metadata.

**Table 6:** Bioinformatic tools.

Bioinformatic tools	No. of responses	%(*n* = 70)
Included in the survey options		
Nextstrain/Nextclade	43	61.4
GISAID EPIFLU/EPICOV	42	60.0
MinKNOW	37	52.9
BaseSpace	35	50.0
EPI2ME	28	40.0
MEGA	24	34.3
Terra.bio	22	31.4
Galaxy	18	25.7
Center for genomic epidemiology (CGE) tools	17	24.3
IRMA	17	24.3
IGV	15	21.4
BioEdit	15	21.4
Geneious	14	20.0
MIRA	14	20.0
FluSurver	13	18.6
Other	13	18.6
CLC Genomics Workbench	11	15.7
BioNumerics	11	15.7
DNAStar	3	4.3
EDGE bioinformatics	3	4.3
Ion reporter server system	2	2.9
Other tools reported as free text by at least 2 respondents		
In-house pipelines	3	4.3
CZ-ID	2	2.9
Genome detective	2	2.9

### 3.9 Funding and sustainability

**Figure 7:**
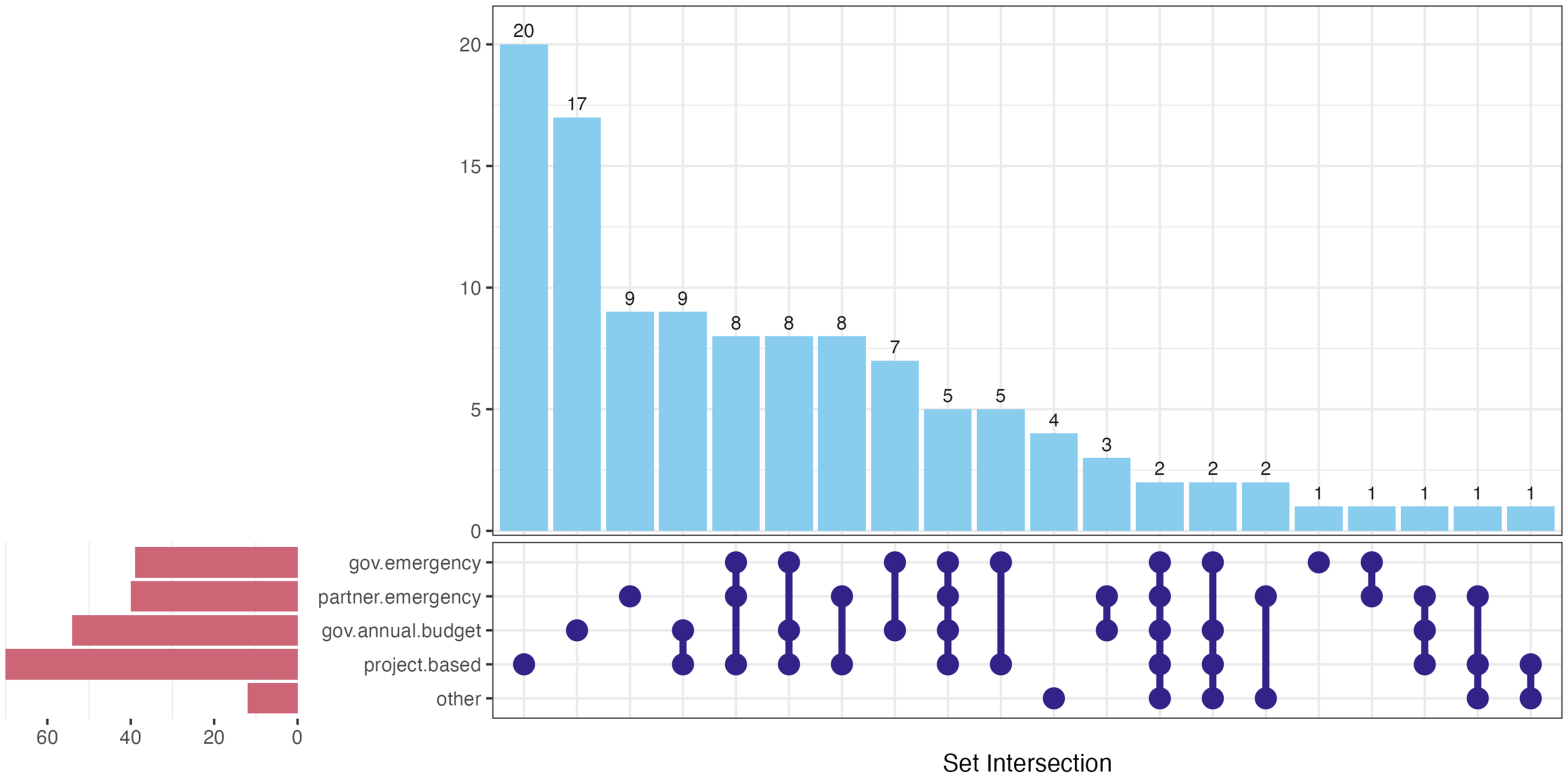
UpSet plot displaying the distribution of funding sources among 113 respondents.

A total of 113 respondents (94.2%) answered questions on laboratory funding sources (Figure 7). The responses indicate that 54.4% (*n* = 62) of respondents receive NGS funding from multiple sources. In contrast, 44.7% (*n* = 51) rely on a single funding source, with one of these being fee-for-service laboratories. Only 17 respondents (14.9%) were solely supported by their governments’ annual budgets, all of which are either HICs or UMICs.

About half of the respondents (63/113) indicated support for NGS activities through emergency funding, whether from their government or a partner, with emergency funding being the sole source of NGS funding for 10 (8.8%) respondents. Project-based funding supported 69 respondents (61.1%). Out of the 46 that responded to the funding sustainability question, 21 (45.6%) indicated availability of long-term funding (of at least 3 years). Of 113 respondents, 53 (46.9%) received government annual budgets for sequencing activities, and 31 of these (31/53, 58.5%) were from HICs. The respondents from LICs (*n* = 5) only reported emergency funding from partners/agencies and research or project-based funding. A detailed breakdown of funding sources is provided in Supplementary File 7.

## 4 Discussion

The GCT 2.0 enables comprehensive costing for sequencing workflows, including equipment, reagents and consumables, personnel, facility, transportation, bioinformatics, and quality management. The global sequencing landscape survey highlighted variability in annual throughput, pathogens, instrumentation, data storage, and funding sources. These variabilities underscore the need for a flexible, comprehensive costing tool that is accessible and usable for laboratories worldwide to support sustainable genomic sequencing activities. While previous studies focus on pathogen-specific activities, or regional capacity, the survey results presented here provide a baseline for both GCT 2.0 development and for broader investment planning and insight into global genomic surveillance.

### 4.1 Sequencing instrumentation

The landscape survey demonstrated the global distribution of sequencing technologies. The survey reiterated that Illumina and ONT are the most widely used sequencing technologies, but the addition of Thermo Fisher and MGI technologies broadens the tool’s application to additional users. Additionally, allowing the user to customize the platform or instrument provides flexibility for the quickly changing landscape. The median number of sequencing instruments per respondent was highest for LMICs, followed by LICs. This may be due to the influx of donated instruments during the COVID-19 pandemic response. Further research may be needed to understand the presence of these sequencing instruments versus their utilization in laboratories in the post-SARS-CoV-2 era, to inform their cost-efficient use and sustainability.

### 4.2 Sequencing loading capacity

In addition to platform and instrument presence in laboratories, differences in instrument-loading optimization further illustrate disparities in operational efficiency across settings. Illumina instruments were more frequently loaded to full capacity (48.6%) compared to ONT (28.8%). This difference may partly reflect platform-specific operational factors. ONT flow cells can be washed and reused in some cases, potentially reducing the incentive to maximize sample loading in a single run. In contrast, Illumina offers a wider range of sequencing kits with varying throughput capacities, allowing laboratories to select kits that better align with pathogen targets and expected sample volumes, whereas ONT platforms have more fixed loading capacities due to a more limited range of flow cell options. Other likely bottlenecks to sequencing run loading optimization include funding, access to specimens, and availability of reagents. Increased throughput may require further resources (automation) for sample prep, data analysis, reagent supply, and/or trained personnel, rather than instruments alone. Additionally, laboratories may not be fully utilizing multiplexing (sequencing multiple samples within a single run) capabilities during sequencing runs, either due to operational constraints or limited awareness of this functionality, resulting in inefficient use of sequencing kits and suboptimal instrument utilization. Cost clarity through the GCT 2.0 may emphasize the financial impact of underloaded runs or the financial benefit of filling runs. The GCT 2.0 allows users to compare the platform and loading capacity impact on costs.

### 4.3 Library preparation kits

The survey results indicated that a variety of library preparation kits are utilized for global sequencing efforts. These varying results reinforce the importance of incorporating customizable kit parameters within GCT 2.0 to ensure accurate, context-specific cost estimation. Such flexibility is essential to maintain the tool’s long-term relevance and applicability across diverse laboratories and regional settings.

### 4.4 Sequencing throughput

The first edition of the GCT provided users with a discrete list of pathogen throughputs, ranging from 600 to 12,000. The landscape survey revealed that throughput from global laboratories varies significantly, from below 100 to over 50,000 samples per year. With over 55% of respondents sequencing fewer than 1,000 samples annually, and 10% looking to establish sequencing capacity, the survey suggests that many facilities may still be in early or intermediate stages of sequencing implementation. As expected, HICs demonstrated the highest mean annual throughput (11,805 samples), compared with 4,002 in LMICs and 910 in LICs, reflecting disparities in infrastructure, human resources, and resource allocation. These findings reiterate the need for accurate and flexible costing to support global laboratories for the implementation, scale-up, and maintenance of sequencing activities. Sample throughput mirrors economic disparity and reflects uneven access to specimens and operational funding. LMICs report fewer Illumina instruments (an average of 1.95 instruments per laboratory), but more ONT instruments (an average of 5.3 instruments per laboratory), possibly due to the cheaper instrument costs, smaller footprint, and often quicker sequencing turnaround time of ONT platforms. It is essential to ensure lower throughputs are covered in the GCT, even though this increases the total cost per sample. The wide-ranging variability underscores the need for custom throughput rather than preset increments for the most accurate costing. Finally, the first edition of the GCT assumed 100% loading capacity for each sequencing run. GCT 2.0 allows users to customize loading capacity to accurately reflect total quantities, costs, and resource utilization.

### 4.5 Pathogens

While the first GCT focused on SARS-CoV-2 sequencing, the survey results captured the varying breadth of sequencing activities globally. The most frequently sequenced pathogens reported were SARS-CoV-2, Influenza viruses and enteric bacteria, followed by HIV, HAIs, or arboviruses.

There was a diverse “Other” category (Supplementary File 6), suggesting emerging needs but limited capacity to expand the focus. GCT 2.0 ensures flexible usability by focusing on a broad range of pathogens, sequencing platforms, and technologies.

Viral sequencing was generally more commonly reported than bacterial sequencing in the responding labs, particularly in HICs. The capacity to respond to outbreaks or conduct broad surveillance is constrained in lower-income regions.

The range of pathogens sequenced highlights the need for a versatile tool rather than a tool designed for specific pathogens. To optimize the global impact of the tool, GCT 2.0 adaptable to diverse global needs. Additionally, GCT 2.0 remains flexible to accommodate multiple pathogen or sequencing targets within one laboratory, enabling accurate costing of multi-pathogen programmes (such as PulseNet for foodborne illnesses).

### 4.6 Data management and analysis

Results from data storage and backup indicate that data integrity and traceability are at risk, particularly in regions lacking robust digital infrastructure. Many labs rely on cloud-based tools or external partners, and internet bandwidth remains a bottleneck (7 labs report download speeds <10 Mbps). Bioinformatics infrastructure remains a significant bottleneck; many lower-resource labs rely on shared or underpowered machines, which are not suited for intensive genome assembly or analysis. Even with sequencing capability, labs may be unable to perform timely analysis, limiting the use of the data to inform public health decision-making, particularly in areas with limited internet access or local expertise. With only 67.5% of respondents indicating that data backup systems are in place, this suggests gaps in long-term data storage capacity.

While there is no formal way to estimate the cost of data loss from poor data management, a comprehensive costing tool should encourage users to consider all costs associated with genomic data generation, analysis, and maintenance. GCT 2.0 is built to quantify both the per-sample costs of NGS data generation and the annual expenditures associated with sequencing activities included in the costing analysis. Without effective data management and reliable backup systems, these investments remain vulnerable to loss.

### 4.7 Funding

The results underscore the variability and complexity of funding landscapes for genomics activities across laboratories. Many laboratories receive support from multiple funding sources, while others rely on a single source of funding, including government allocations and fee-for-service income. Notably, laboratories solely funded through annual government budgets were all situated in high-income or upper-middle-income countries, reflecting disparities in national investment capacity. In lower-resource settings, limited access to sustained or diversified funding can create significant barriers, particularly when funders require detailed expense justification. These constraints may also affect the long-term sustainability of genomics services and reduce cost-efficiency, as laboratories operating with limited budgets may sequence smaller batches of samples, resulting in higher per-sample costs, as reflected in the sequencing loading capacity and sequencing throughput findings described above.

LICs reported the highest median number of sequencing instruments per laboratory; however, these instruments are often significantly underutilized, revealing a critical disconnect between infrastructure availability and operational capacity. This underuse is closely tied to funding constraints, as LICs mentioned relying exclusively on emergency-based support, which is typically short-term and reactive. Oftentimes, sequencing instruments may be donated to a laboratory for a single sequencing programme, without further resources to sustain the activity. This funding model undermines the continuity and effectiveness of genomic surveillance efforts. To fully leverage existing infrastructure, there is an urgent need for coordinated, long-term strategies that prioritize sustainable financing, workforce development, cost-efficient set-ups, and institutional capacity building. The GCT 2.0 is critical to strategy development and understanding of financial commitments.

With approximately half of respondents indicating funding from pandemic response, there is a clear need to support laboratory costing for sustainable activities. Resources and equipment purchased during the pandemic response will require continued upkeep, funding, and utilization.

### 4.8 Survey findings and implications for GCT 2.0

The survey findings presented here provide a clear empirical basis for the design decisions embedded in GCT 2.0. Low annual throughputs across the majority of responding laboratories supported the replacement of fixed throughput increments with a fully customizable input. The widespread use of ONT, Thermo Fisher, and MGI platforms alongside Illumina justified expanding platform coverage beyond the original two. The diversity of pathogens sequenced globally drove the shift from a SARS-CoV-2-specific tool to one supporting a broad range of viral and bacterial pathogens, including multi-pathogen programmes.

In addition, suboptimal sequencer loading across income settings informed the decision to base costing on average rather than maximum run loading, with loading capacity surfaced as a prompt for cost-efficiency review. The variety of library preparation kits reported in use reinforced the need for customizable, fully integrated reagent inputs. Finally, the fragmented and largely emergency-driven funding landscape underscored the need for a tool that can model diverse funding scenarios and support laboratories in communicating the full, ongoing costs of sustainable genomic surveillance programmes.

### 4.9 Limitations of the study

This study has some limitations. The survey followed a convenience sampling and snowball approach, leading to limitations in outreach and results, particularly in some regions. As a cross-sectional, self-reported online survey, responses reflect a single time point and may be influenced by self-selection and reporting biases. No survey questions were mandatory; therefore, respondents could omit items, resulting in variable response counts across questions and unknown reasons for nonresponse. Despite broad dissemination and multilingual translation, some regions and institution types may have been underrepresented, and interpretation of technical questions could vary. While data cleaning addressed inconsistencies and anomalies, objective verification of reported capacity, throughput, and infrastructure was not possible. Finally, the survey focused primarily on infectious disease surveillance laboratories, which may limit generalizability to research-focused or non-public health sequencing activities.

## 5 Conclusion and recommendations

Global sequencing capacity is expanding but remains uneven, highlighting persistent inequities and opportunities to address systemic gaps. Capacity constraints extend beyond instrument availability. Disparities in sustainable financing, throughput limitations, underutilization of equipment, limited automation, data storage, and insufficient bioinformatics infrastructure (particularly in low-resource settings) remain significant bottlenecks. Consequently, these constraints may limit the laboratory’s ability to conduct timely, efficient, and accurate genomic surveillance, reinforcing the need for a tool that supports strategic, optimized planning and resource allocation.

Findings from this survey highlight the pressing need for a standardized, detailed, and transparent costing tool that can support laboratories in calculating and communicating the full costs of genomic sequencing activities. Such a tool strengthens funding applications by improving cost transparency and supports more equitable access to financing by enabling laboratories to articulate resource needs more effectively. Beyond funding applications, the GCT 2.0 supports dynamic pricing models and enables cost-recovery calculations, ensuring that the fee-for-service prices charged do not leave the lab in deficit and provide further justification and transparency for service fees. Finally, an open-source tool enables quick cost estimation with minimal bureaucracy, providing opportunities for efficiency and utilization during emergency response or rapid changes in the landscape or laboratory needs.

The second version of the GCT incorporates a modular framework to accommodate funding sources, including emergency response, long-term government budgets, and fee-for-service models. It provides flexible throughput and instrument options, distinguish fixed and variable costs, and capture requirements for data management and bioinformatics capacity. Customizable data fields provide the most accurate costing analysis for tool users. Additionally, embedding sustainability and cost-recovery strategies across disease programs will likely further enhance utility. By offering a comprehensive and adaptable costing framework, GCT 2.0 can help laboratories as well as other stakeholders plan, manage, and sustain sequencing and bioinformatics activities, strengthening global genomic surveillance.

## Data Availability

The original contributions presented in the study are included in the article/Supplementary material, further inquiries can be directed to the corresponding author.

## Supplementary material

SUPPLEMENTARY FILE 1 Survey tool (English).

SUPPLEMENTARY FILE 2 Survey tool (French).

SUPPLEMENTARY FILE 3 Survey tool (Russian).

SUPPLEMENTARY FILE 4 Survey tool (Spanish).

SUPPLEMENTARY FILE 5 Survey solicitation.

SUPPLEMENTARY FILE 6 Summary analysis file.

SUPPLEMENTARY FILE 7 Funding sources for the laboratory stratified by country development level.
