# Supplementary material for: Next-generation sequencing and bioinformatics capacity: findings from a multi-country survey to guide the genomics costing tool 2.0
Source: Front Public Health. 2026 Jun 25;14:1838184. doi: 10.3389/fpubh.2026.1838184 (PMC13367074; doi:10.3389/fpubh.2026.1838184)
Supplement: SUPPLEMENTARY FILE 1 — Survey tool (English). [file Table_1.DOCX]

**Genomics Costing Tool Survey - English**

This information will be utilized to support prioritizing components to be included in an updated version of the [Genomics Costing Tool (GCT)](https://www.who.int/publications/i/item/9789240090866). ([GCT Article)](https://doi.org/10.3389/fpubh.2024.1404243)

Note: The data collected from this survey will be anonymized to ensure that individual responses cannot be traced back to any participant. The anonymized data will then be summarized to highlight key findings and trends. These summarized results may be used in a manuscript that will be submitted for publication in a peer-reviewed journal. By participating in this survey, you consent to the use of your anonymized data for these purposes. Your privacy and confidentiality are of utmost importance to us, and all necessary measures will be taken to protect your information.

1. Laboratory Name
2. Laboratory Country
3. Type of laboratory

National

Sub-national

Other (please specify)

1. Laboratory Point of Contact Email and Phone Number

Email

Phone Number:

1. Which language(s) would be most beneficial for translating the GCT? (Select all that apply):

Arabic

Chinese

English

European Portuguese

French

Russian

Spanish

Other (please specify)

1. What is the source of funding for genomic sequencing? (Select all that apply)

Emergency funding from partners/agencies

Emergency response funds from your government

Government annual budget

Long term (at least 3 years) funding from partner organizations

No long term funding identified

Research or project based funding

Other (please specify)

1. Is the laboratory responsible for costing?

Yes – The laboratory completes costing

No – Costing is completed externally

1. Is the laboratory responsible for procurement?

Yes – The laboratory completes procurement

No – Procurement is completed externally

1. Identify all high-priority pathogens currently under surveillance that require routine genomic sequencing.

SARS-CoV-2

Influenza

Other respiratory viruses

Enteric bacteria

Hospital acquired infection (HAI) pathogens

HIV

HIV drug resistance

Arboviruses

*Mycobacterium tuberculosis* (MTB)

MTB drug resistance

Other (please specify)

1. Input an estimated average annual throughput (number of samples) for each pathogen

*Text boxes appear depending on answers from above

1. What is the current the annual throughput for all pathogens being sequenced?

0 – looking to establish a sequencing laboratory

1-100

101-600

601-1000

1001-2000

2001-3000

4001-5000

5001+

1. Does your lab perform sequencing for non-infectious disease modalities (i.e., oncology, human genetics)

No

Yes, please specify

1. What types of specimens are accepted for sequencing? (Select all that apply)

Human tissue (i.e., lung tissue)

Nasopharyngeal swabs (NP)

Oropharyngeal swabs (OP)

Nasal mid-turbinate (NMT)

Anterior nasal swabs

Nasopharyngeal wash/aspirate or nasal wash/aspirate

Bronchoalveolar lavage

Tracheal aspirate

Pleural fluid

Saliva

Sputum

Stool

Whole blood

Serum

Plasma

Other (please specify)

1. What extraction kit(s) are used for nucleic acid extraction for sequencing? (Select all that apply)

Qiagen Dneasy Blood & Tissue Kits

Qiagen QIAamp DNA Kits

Qiagen EZ1/2 DNA Tissue Kit

Promega Wizard DNA Extraction Kit

Applied Biosystems MagMAX total nucleic acid isolation kit

Other (please specify)

1. Are automated extraction platforms used for extraction?

Yes

No (skip to page 10)

1. What automated extraction platforms are used? (Select all that apply)

Qiagen QIAsymphony

Qiagen EZ2 Connect

Qiagen EZ1 Advanced XL

Qiagen QIAcube Connect/HT

Roche MagnaPure

PerkinElmer Chemagic 360

ThermoFisher Kingfisher

Eppendorf EpMotion 5073t

Eppendorf EpMotion 5075t

Eppendorf EpMotion 5075v

Eppendorf EpMotion 5075vt

Other (please specify)

1. Are liquid handling systems used for automated library preparation?

Yes

No (skip to page 12)

1. What liquid handling devices are available in the sequencing laboratory? (Select all that apply)

Eppendorf EpMotion 5073t

Eppendorf EpMotion 5075t

Promega Maxprep

Beckman Coulter Biomek

PerkinElmer Sciclone

ClearLabs System

Opentrons OT-2

Other (please specify)

1. Which Illumina instruments are available? (Select all that apply)

iSeq

MiniSeq

MiSeq

NextSeq 500

NextSeq 550

NextSeq 1000/2000

None (skip to page 15)

Other (please specify)

1. How many of each Illumina instrumentation are available?

*Text boxes appear to input number depending on answers above

1. Which Illumina sequencing kits are used? (Select all that apply)

iSeq 100 i1 v2

MiniSeq RapidKit

MiniSeq Mid Output (300c)

MiniSeq High Output (75c)

MiniSeq High Output (150c)

MiniSeq High Output (300c)

MiSeq v2 Nano (300c)

MiSeq v2 Nano (500c)

MiSeq v2 Micro (300c)

MiSeq v2 (300c)

MiSeq v2 (500c)

MiSeq v3 (150c)

MiSeq v3 (600c)

NextSeq Mid (150c)

NextSeq Mid (300c)

NextSeq High (75c)

NextSeq High (300c)

NextSeq High (500c)

NextSeq P1 (100c)

NextSeq P1 (300c)

NextSeq P1 (600c)

NextSeq P2 v3 (100c)

NextSeq P2 v3 (200c)

NextSeq P2 v3 (300c)

NextSeq P3 (50c)

NextSeq P3 (100c)

NextSeq P3 (200c)

NextSeq P3 (300c)

NextSeq P4 (50c)

NextSeq P4 (100c)

NextSeq P4 (200c)

NextSeq P4 (300c)

NextSeq P4 (50c)

Other (please specify)

1. Which library preparation kits are used for Illumina sequencing? (Select all that apply)

NexteraXT

Illumina DNA Prep

Illumina COVIDSeq

Illumina Respiratory Virus Oligo Panel

Illumina Respiratory Pathogen ID/AMR Enrichment Panel Kit

Other (please specify)

1. Are Illumina runs loaded to full capacity?

Yes – Loading capacity always optimized

Sometimes – Loading capacity is sometimes optimized and sometimes under loaded

No – Loading capacity is not optimized, sample volume frequency too low for loading capacity optimization

1. Are the Illumina sequencing instruments shared with any other lab groups or is it dedicated to your lab group?

Yes - shared

No - dedicated

Other (please specify)

1. Which ONT sequencing instrumentation is available? (Select all that apply)

MinION Mk1C

MinION Mk1D

MinION Mk1B

GridION

PromethION (including P2 and P2 Solo)

None (skip to page 18)

Other (please specify)

1. How many of each ONT instrumentation are available?

*Text boxes appear to input number depending on answers above

1. What library preparation kits are used for ONT sequencing? (Select all that apply)

Ligation Sequencing Kit V14 (SQK-LKS114)

16S Barcoding Kit 1-24 (SQK-16S023)

Rapid Sequencing Kit V14 (SQK-RAD114)

Ultra-Long DNA Sequencing Kit V14 (SQK-ULK114)

Rapid PCR Barcoding Kit (SQK-RPB004)

Midnight RT PCR Expansion (EXP-MRT001)

Rapid Barcoding Kit (SQK-RBK110.96)

Other (please specify)

1. Is ONT sequencing instrumentation shared among groups?

Yes

No

Other (please specify)

1. Are ONT runs loaded to full capacity?

Yes – Loading capacity always optimized

Sometimes – Loading capacity is sometimes optimized and sometimes under loaded

No – Loading capacity is not optimized, sample volume frequency too low for loading capacity optimization

1. Which Thermo Fisher sequencing instruments are available? (Select all that apply)

Ion Chef™ Instrument

Ion OneTouch™ 2 System

Ion OneTouch™ 2 Instrument

Service Plan for Ion Torrent™ Next-Generation Sequencing System

Ion GeneStudio S5 System

Ion GeneStudio S5 Plus System

Ion GeneStudio S5 Prime System

Ion PGM Dx System

Ion Torrent Genexus System

Genexus Purification System

Genexus Integrated Sequencer

None (skip to page 21)

Other (please specify)

1. How many of each Thermo Fisher instrumentation are available?

*Text boxes appear to input number depending on answers above..

1. Which Thermo Fisher sequencing kits are used?

Ion PGM™ Template OT2 400 Kit

Ion PI™ IC 200 Kit

Ion PI™ Template OT2 200 Kit v2

Ion PI™ Template OT2 200 Kit v3

Ion S5™ Calibration Standard

Ion PGM™ 200 Sequencing Kit

Ion PGM™ Sequencing 400 Kit

Ion PI™ Sequencing 200 Kit v2

Ion PI™ Sequencing 200 Kit v3

Other (please specify)

1. Which library preparation kits are used for Thermo Fisher sequencing?

Ion AmpliSeq™ Library Kit 2.0

Ion AmpliSeq™ RNA Library Kit

Ion TargetSeq™ Custom Enrichment Kit, 100-500 kb

Ion TargetSeq™ Custom Enrichment Kit, 500 kb-2 Mb

Ion TargetSeq™ Custom Enrichment Kit, 2-10 Mb

Ion Xpress™ Plus Fragment Library Kit

Ion Plus Fragment Library Kit

Thermo Scientific® MuSeek™ Library Preparation Kit for the Ion Torrent™ instrument

NEBNext® Fast DNA Fragmentation & Library Prep Set for Ion Torrent

NEBNext® Fast DNA Library Prep Set for Ion Torrent 4

Ion Xpress™ Plus Fragment Library Kit for AB Library Builder™ System

Ion Plus Fragment Library Kit for AB Library Builder™ System

Ion 16S™ Metagenomics Kit

Ion TrueMate™ Library Kit

Ion TrueMate™ Plus Library Kit

Ion Total RNA-Seq Kit v2

Magnetic Bead Purification Module

Ion Plus Fragment Library Adapters

Ion Xpress™ RNA-Seq Barcode 1-16 Kit

Ion Xpress™ Barcode Adapters 1-16 Kit

Ion Xpress™ Barcode Adapters 17-32 Kit

Ion Xpress™ Barcode Adapters 33-48 Kit

Ion Xpress™ Barcode Adapters 49-64 Kit

Ion Xpress™ Barcode Adapters 65-80 Kit

Ion Xpress™ Barcode Adapters 81-96 Kit

Ion Xpress™ Barcode Adapters 1-96 Kit

Other (please specify)

1. Are Thermo Fisher runs loaded to full capacity?

Yes – Loading capacity always optimized

Sometimes – Loading capacity is sometimes optimized and sometimes under loaded

No – Loading capacity is not optimized, sample volume frequency too low for loading capacity optimization

1. Are the Thermo Fisher sequencing instruments shared with any other lab groups or is it dedicated to your lab group?

Yes - shared

No - dedicated

Other (please specify)

1. Which MGI sequencing instruments are available? ((Select all that apply) DNBSEQ-T7

DNBSEQ-G400

DNBSEQ-G50

DNBSEQ-G99

DNBSEQ-E25

None (skip to page 24)

Other (please specify)

1. How many of each MGI instrumentation are available?

*Text boxes appear to input number depending on answers above

1. Which MGI library preparation kits are used? (Select all that apply)

MGIEasy Fast PCR-FREE FS Library Prep Set V2.0

MGIEasy Fast FS Library Prep Set V2.0

MGIEasy Duplex UMI Universal Library Prep Set

MGIEasy UDB Universal Library Prep Set

MGIEasy Fast RNA Library Prep Set

MGIEasy PCR-Free DNA Library Prep Set

MGIEasy FS PCR-Free DNA Library Prep Set

MGIEasy RNA Library Prep Set

MGIEasy FS DNA Library Prep Set

MGIEasy Universal DNA Library Prep Set

MGIEasy Fast FS DNA Library Prep Set

MGIEasy Respiratory Microorganism Genome Library Preparation Set

Other (please specify)

1. Which sequencing kits are used for MGI sequencing? (Select all that apply)

DNBSEQ-G400 High-throughput Sequencing Set

DNBSEQ-G400 High-throughput Rapid Sequencing Set

DNBSEQ-G50RS High-throughput (Rapid) Sequencing Set

DNBSEQ G99 High-throughput Sequencing Set

DNBSEQ-T7RS High-throughput Sequencing Set

Other (please specify)

1. Are MGI runs loaded to full capacity?

Yes – Loading capacity always optimized

Sometimes – Loading capacity is sometimes optimized and sometimes under loaded

No – Loading capacity is not optimized, sample volume frequency too low for loading capacity optimization

1. Are the MGI instruments shared with any other lab groups or is it dedicated to your lab group?

Yes - shared

No - dedicated

Other (please specify)

1. Please select any other sequencing instrumentation available in the laboratory (Select all that apply)

Sanger sequencing - ABI Genetic Analyzer

Sanger sequencing - Promega Spectrum

Ultima Genomics

Element Biosciences

Pacific Bio (PacBio)

None

Other (please specify)

1. Please select instrumentation available for quality control of the sequencing process to the laboratory. (Select all that apply)

Qubit fluorometer

Nanodrop

Fluorescent plate reader

Fragment Analyzer (i.e., Bioanalyzer or TapeStation)

None

Other (please specify)

1. Does the lab have access to a computer(s) specifically for bioinformatic analysis of sequencing data?

Yes

No

1. What is the internet upload/download speed? ([Test here](https://fast.com/))

Upload less than 10 mbps

Upload more than 10mbps

Download less than 10mbps

Download more than 10mpbs

1. Which of the following bioinformatic tools are utilized?

BaseSpace

EPI2ME

MinKNOW

CGE Tools (ResFinder, VirulenceFinder, PlasmidFinder, SerotypeFinder)

Nextstrain/Nextclade

FluServer

GISAID EPIFLU/EPICOV

Terra.bio

CLC Genomics

BioNumerics

Geneious

IGV

IRMA

MIRA

DNAStar

BioEdit

EDGE

MEGA

Galaxy

Ion Reporter Server System

Ubuntu Operating System

Other (please specify)

None of the above

1. Where is sequencing data stored long term? (Select all that apply)

File server

External hard drive

Computer hard disk

Cloud storage

Other

1. Is there a backup of sequencing data storage?

Yes

No

1. Where is sequence data backed-up?

File server

External hard drive

Computer hard disk

Cloud storage

None

Other

1. Is there a LIMS database to link sequences to metadata?

Yes

No
