# Supplementary material for: Next-generation sequencing and bioinformatics capacity: findings from a multi-country survey to guide the genomics costing tool 2.0
Source: Front Public Health. 2026 Jun 25;14:1838184. doi: 10.3389/fpubh.2026.1838184 (PMC13367074; doi:10.3389/fpubh.2026.1838184)
Supplement: SUPPLEMENTARY FILE 2 — Survey tool (French). [file Table_2.DOCX]

**Enquête sur les outils d'évaluation des coûts de la génomique - Français**

Ces données seront utilisées pour aider à hiérarchiser les intrants à inclure dans une version actualisée de l'[outil d'évaluation des coûts de la génomique](https://www.who.int/publications/i/item/9789240090866) (GCT) ([article GCT)](https://doi.org/10.3389/fpubh.2024.1404243).

Note : Les données collectées dans le cadre de cette enquête seront anonymisées afin de garantir que les réponses individuelles ne permettent pas de remonter jusqu'à un participant. Les données anonymes seront ensuite résumées afin de mettre en évidence les principaux résultats et tendances. Ces résultats résumés pourront être utilisés dans un manuscrit qui sera soumis pour publication dans un journal scientifique. En participant à cette enquête, vous acceptez que vos données anonymes soient utilisées à ces fins. Toutes les mesures nécessaires seront prises pour protéger vos informations privées et votre confidentialité.

1. Nom du laboratoire
2. Laboratoire Pays
3. Type de laboratoire

Nationales

Infranational

Autre (à préciser)

1. Courriel et numéro de téléphone du point de contact du laboratoire

Courriel

Numéro de téléphone :

1. Le GCT devrait être disponible dans quelle(s) langue(s) ? (Sélectionnez toutes les réponses qui s'appliquent) :

L'Arabe

Chinois

Anglais

Portugais Français

Russie

Espagnol

Autre (à préciser)

1. Quelle est la source de financement du programme de séquençage génomique dans votre laboratoire ? (Sélectionnez toutes les réponses qui s'appliquent)

Financement d'urgence par les partenaires/agences

Fonds d'intervention d'urgence de votre gouvernement

Budget annuel du gouvernement

Financement à long terme (au moins 3 ans) par des organisations partenaires

Pas de financement à long terme identifié

Financement de la recherche ou de projets

Autre (à préciser)

1. Le laboratoire est-il responsable du calcul des coûts ?

Oui - Le laboratoire procède à l'évaluation des coûts

Non - Le calcul des coûts est effectué en externe

1. Le laboratoire est-il responsable de l'approvisionnement ?

Oui - Le laboratoire effectue les achats

Non - La passation des marchés est effectuée en externe

1. Identifier tous les agents pathogènes prioritaires actuellement sous surveillance qui nécessitent un programme de séquençage génomique.

SARS-CoV-2

Grippe

Autres virus respiratoires

Bactéries entériques

Agents pathogènes des infections nosocomiales (IHN)

VIH

Résistance du VIH aux médicaments

Arbovirus

*Mycobacterium tuberculosis* (MTB)

Résistance aux médicaments contre la tuberculose

Autre (à préciser)

1. Pour chaque agent pathogène , indiquer une moyenne annuelle du nombre d'échantillons .

*Les boîtes de texte apparaissent en fonction des réponses ci-dessus.

1. Quel est le nombre annuel d’échantillons séquencés pour tous les agents pathogènes ?

0 - cherche à établir un laboratoire de séquençage

1-100

101-600

601-1000

1001-2000

2001-3000

4001-5000

5001+

1. Votre laboratoire réalise-t-il du séquençage pour maladies non infectieuses (par exemple, oncologie, génétique humaine) ?

Non

Oui, veuillez préciser

1. Quels types de spécimens sont acceptés pour le séquençage ? (Sélectionnez toutes les réponses qui s'appliquent)

Tissu humain (par exemple, tissu pulmonaire)

Écouvillons nasopharyngés (NP)

Écouvillons oropharyngés (OP)

Turbine nasale moyenne (NMT)

Écouvillons nasaux antérieurs

Lavage/aspiration nasopharyngé(e) ou lavage/aspiration nasal(e)

Lavage broncho-alvéolaire

Aspiration trachéale

Liquide pleural

Salive

Expectorations

Tabouret

Sang total

Sérum

Plasma

Autre (à préciser)

1. Quel(s) kit(s) d'extraction sont utilisés pour l'extraction de l'acide nucléique pour le séquençage ? (Sélectionnez toutes les réponses qui s'appliquent)

Kits Qiagen Dneasy pour le sang et les tissus

Kits d'ADN QIAamp de Qiagen

Kit tissu ADN EZ1/2 de Qiagen

Kit d'extraction d'ADN Wizard de Promega

Kit d'isolation de l'acide nucléique total MagMAX d'Applied Biosystems

Autre (à préciser)

1. Des plateformes d'extraction automatisées sont-elles utilisées pour l'extraction ?

Oui

Non (passez à la page 10)

1. Quelles sont les plateformes d'extraction automatisées utilisées ? (Sélectionnez toutes les réponses qui s'appliquent)

Qiagen QIAsymphony

Qiagen EZ2 Connect

Qiagen EZ1 Advanced XL

Qiagen QIAcube Connect/HT

Roche MagnaPure

PerkinElmer Chemagic 360

ThermoFisher Kingfisher

Eppendorf EpMotion 5073t

Eppendorf EpMotion 5075t

Eppendorf EpMotion 5075v

Eppendorf EpMotion 5075vt

Autre (à préciser)

1. Des systèmes de manipulation des liquides sont-ils utilisés pour la préparation automatisée des librairies ?

Oui

Non (passez à la page 12)

1. Quels sont les dispositifs de manipulation des liquides disponibles dans le laboratoire de séquençage ? (Sélectionnez tous les éléments qui s'appliquent)

Eppendorf EpMotion 5073t

Eppendorf EpMotion 5075t

Promega Maxprep

Beckman Coulter Biomek

PerkinElmer Sciclone

Système ClearLabs

Opentrons OT-2

Autre (à préciser)

1. Quels sont les instruments Illumina disponibles ? (Sélectionnez tous ceux qui s'appliquent. Veuillez préciser le nombre de chaque instrument dans la section des commentaires

iSeq

MiniSeq

MiSeq

NextSeq 500

NextSeq 550

NextSeq 1000/2000

Aucun (passez à la page 15)

Autre (à préciser)

1. Combien de chaque instrument Illumina sont disponibles ?

*Des boîtes de texte apparaissent pour saisir le nombre en fonction des réponses ci-dessus.

1. Quels kits de séquençage Illumina sont utilisés ? (Sélectionnez tout ce qui s'applique)

iSeq 100 i1 v2

MiniSeq RapidKit

MiniSeq Mid Output (300c)

MiniSeq High Output (75c)

MiniSeq High Output (150c)

MiniSeq High Output (300c)

MiSeq v2 Nano (300c)

MiSeq v2 Nano (500c)

MiSeq v2 Micro (300c)

MiSeq v2 (300c)

MiSeq v2 (500c)

MiSeq v3 (150c)

MiSeq v3 (600c)

NextSeq Mid (150c)

NextSeq Mid (300c)

NextSeq High (75c)

NextSeq High (300c)

NextSeq High (500c)

NextSeq P1 (100c)

NextSeq P1 (300c)

NextSeq P1 (600c)

NextSeq P2 v3 (100c)

NextSeq P2 v3 (200c)

NextSeq P2 v3 (300c)

NextSeq P3 (50c)

NextSeq P3 (100c)

NextSeq P3 (200c)

NextSeq P3 (300c)

NextSeq P4 (50c)

NextSeq P4 (100c)

NextSeq P4 (200c)

NextSeq P4 (300c)

NextSeq P4 (50c)

Autre (à préciser)

1. Quels kits de préparation de librairies sont utilisés pour le séquençage Illumina ? (Sélectionnez toutes les réponses qui s'appliquent)

NexteraXT

Préparation de l'ADN d'Illumina

Illumina COVIDSeq

Panel d'oligo-virus respiratoires Illumina

Kit d'enrichissement Illumina Respiratory Pathogen ID/AMR Panel

Autre (à préciser)

1. Les analyses avec séquenceurs Illumina sont-elles chargées à pleine capacité ?

Oui - La capacité de chargement est toujours optimisée

Parfois - La capacité de chargement est parfois optimisée et parfois sous-chargée

Non - La capacité de chargement n'est pas optimisée, la fréquence du volume de l'échantillon est trop faible pour permettre l'optimisation de la capacité de chargement.

1. Les séquenceurs Illumina sont-ils partagés avec d'autres groupes de laboratoire ou sont-ils réservés à votre groupe de laboratoire ?

Oui - partagé

Non - dédié

Autre (à préciser)

1. Avez-vous des séquenceurs Nanopore dans votre laboratoire ? (Sélectionnez tout ce qui s'applique)

MinION Mk1C

MinION Mk1D

MinION Mk1B

GridION

PromethION (y compris P2 et P2 Solo)

Aucun (passez à la page 18)

Autre (à préciser)

1. Combien e chaque instrument Nanopore sont disponibles ?

*Des boîtes de texte apparaissent pour saisir le nombre en fonction des réponses ci-dessus.

1. Quels kits de préparation de librairies sont utilisés pour le séquençage ONT ? (Sélectionnez toutes les réponses qui s'appliquent)

Kit de séquençage par ligature V14 (SQK-LKS114)

Kit de codage à barres 16S 1-24 (SQK-16S023)

Kit de séquençage rapide V14 (SQK-RAD114)

Kit de séquençage d'ADN ultra-long V14 (SQK-ULK114)

Kit de codage rapide par PCR (SQK-RPB004)

Expansion Midnight RT PCR (EXP-MRT001)

Kit de codage rapide (SQK-RBK110.96)

Autre (à préciser)

1. Les séquenceurs ONT sont-ils partagés avec d'autres groupes de laboratoire ou sont-ils réservés à votre groupe de laboratoire ?

Oui

Non

Autre (à préciser)

1. Les analyses avec séquenceurs ONT sont-elles chargées à pleine capacité ?

Oui - La capacité de chargement est toujours optimisée

Parfois - La capacité de chargement est parfois optimisée et parfois sous-chargée

Non - La capacité de chargement n'est pas optimisée, la fréquence du volume de l'échantillon est trop faible pour permettre l'optimisation de la capacité de chargement.

1. Les séquenceurs Thermo Fisher sont-ils partagés avec d'autres groupes de laboratoire ou sont-ils réservés à votre groupe de laboratoire ? (Sélectionnez tout ce qui s'applique)

Instrument Ion Chef™

Système Ion OneTouch™ 2

Instrument Ion OneTouch™ 2

Plan de service pour le système de séquençage de nouvelle génération Ion Torrent™

Système Ion GeneStudio S5

Système Ion GeneStudio S5 Plus

Système Ion GeneStudio S5 Prime

Système Ion PGM Dx

Système Genexus de Ion Torrent

Système de purification Genexus

Séquenceur intégré Genexus

Aucun (passez à la page 21)

Autre (à préciser)

1. Combien e chaque instrument Thermo Fisher sont disponibles ?

*Des boîtes de texte apparaissent pour saisir un nombre en fonction des réponses ci-dessus...

1. Quels kits de séquençage Thermo Fisher sont utilisés ?

Kit Ion PGM™ Template OT2 400

Kit Ion PI™ IC 200

Ion PI™ Template OT2 200 Kit v2

Ion PI™ Template OT2 200 Kit v3

Etalon d'étalonnage Ion S5™

Kit de séquençage Ion PGM™ 200

Kit Ion PGM™ Sequencing 400

Ion PI™ Sequencing 200 Kit v2

Ion PI™ Sequencing 200 Kit v3

Autre (à préciser)

1. Quels kits de préparation de librairies sont utilisés pour le séquençage Thermo Fisher ?

Kit de librairies Ion AmpliSeq™ 2.0

Kit de librairies d'ARN Ion AmpliSeq™

Kit d'enrichissement personnalisé Ion TargetSeq™, 100-500 kb

Kit d'enrichissement personnalisé Ion TargetSeq™, 500 kb-2 Mb

Kit d'enrichissement personnalisé Ion TargetSeq™, 2-10 Mb

Kit de librairie de fragments Ion Xpress™ Plus

Kit de librairie de fragments Ion Plus

Kit de préparation de librairies Thermo Scientific® MuSeek™ pour l'instrument Ion Torrent™.

NEBNext® Fast DNA Fragmentation & Library Prep Set pour Ion Torrent

NEBNext® Fast DNA Library Prep Set pour Ion Torrent 4

Kit de librairie de fragments Ion Xpress™ Plus pour le système AB Library Builder™

Kit de librairie de fragments Ion Plus pour le système AB Library Builder™

Kit Ion 16S™ Metagenomics

Kit de librairies Ion TrueMate™

Kit de librairies Ion TrueMate™ Plus

Kit Ion Total RNA-Seq v2

Module de purification par billes magnétiques

Adaptateurs pour librairies de fragments Ion Plus

Kit Ion Xpress™ RNA-Seq Barcode 1-16

Kit d'adaptateurs de code-barres Ion Xpress™ 1-16

Kit d'adaptateurs de code-barres Ion Xpress™ 17-32

Kit d'adaptateurs de code-barres Ion Xpress™ 33-48

Kit d'adaptateurs de code-barres Ion Xpress™ 49-64

Kit d'adaptateurs de code-barres Ion Xpress™ 65-80

Kit d'adaptateurs de code-barres Ion Xpress™ 81-96

Kit d'adaptateurs de code-barres Ion Xpress™ 1-96

Autre (à préciser)

1. Les analyses avec séquenceurs de Thermo Fisher sont-ils chargés à pleine capacité ?

Oui - La capacité de chargement est toujours optimisée

Parfois - La capacité de chargement est parfois optimisée et parfois sous-chargée

Non - La capacité de chargement n'est pas optimisée, la fréquence du volume de l'échantillon est trop faible pour permettre l'optimisation de la capacité de chargement.

1. Les Thermo Fisher sont-ils partagés avec d'autres groupes de laboratoire ou sont-ils réservés à votre groupe de laboratoire ?

Oui - partagé

Non - dédié

Autre (à préciser)

1. Avez-vous des séquenceurs Nanopore dans votre laboratoire ? (Sélectionnez tout ce qui s'applique)

DNBSEQ-T7

DNBSEQ-G400

DNBSEQ-G50

DNBSEQ-G99

DNBSEQ-E25

Aucun (passez à la page 24)

Autre (à préciser)

1. Combien de chaque instrument MGI sont disponibles ?

*Des boîtes de texte apparaissent pour saisir le nombre en fonction des réponses ci-dessus.

1. Quels kits de préparation de librairies MGI sont utilisés ? (Sélectionnez toutes les réponses qui s'appliquent)

MGIEasy Fast PCR-FREE FS Library Prep Set V2.0

MGIEasy Fast FS Library Prep Set V2.0

MGIEasy Duplex UMI Universal Library Prep Set (Kit de préparation de librairie universelle MGIEasy Duplex UMI)

MGIEasy UDB Universal Library Prep Set (Kit de préparation de librairie universelle MGIEasy UDB)

MGIEasy Fast RNA Library Prep Set (Kit de préparation de librairies d'ARN rapide MGIEasy)

MGIEasy PCR-Free DNA Library Prep Set (Kit de préparation de librairies d'ADN sans PCR)

MGIEasy FS PCR-Free DNA Library Prep Set (Kit de préparation de librairies d'ADN sans PCR)

MGIEasy RNA Library Prep Set

MGIEasy FS DNA Library Prep Set

MGIEasy Universal DNA Library Prep Set

MGIEasy Fast FS DNA Library Prep Set (Kit de préparation de librairies d'ADN)

MGIEasy Respiratory Microorganism Genome Library Preparation Set (Kit de préparation de librairies de génomes de micro-organismes respiratoires MGIEasy)

Autre (à préciser)

1. Quels kits de séquençage sont utilisés pour le séquençage MGI ? (Sélectionnez toutes les réponses qui s'appliquent)

DNBSEQ-G400 Set de séquençage à haut débit

DNBSEQ-G400 Set de séquençage rapide à haut débit

DNBSEQ-G50RS Set de séquençage à haut débit (rapide)

Jeu de séquençage à haut débit DNBSEQ G99

DNBSEQ-T7RS Set de séquençage à haut débit

Autre (à préciser)

1. Les analyses avec séquenceurs MGI sont-ils chargés à pleine capacité ?

Oui - La capacité de chargement est toujours optimisée

Parfois - La capacité de chargement est parfois optimisée et parfois sous-chargée

Non - La capacité de chargement n'est pas optimisée, la fréquence du volume de l'échantillon est trop faible pour permettre l'optimisation de la capacité de chargement.

1. Les instruments MGI sont-ils partagés avec d'autres groupes de laboratoire ou sont-ils réservés à votre groupe de laboratoire ?

Oui - partagé

Non - dédié

Autre (à préciser)

1. Veuillez sélectionner tout autre instrument de séquençage disponible dans votre laboratoire (Sélectionnez toutes les réponses qui s'appliquent)

Séquençage Sanger - Analyseur génétique ABI

Séquençage Sanger - Promega Spectrum

Ultima Genomics

Element Biosciences

Pacific Bio (PacBio)

Aucun

Autre (à préciser)

1. Veuillez sélectionner les instruments disponibles pour le contrôle de la qualité du processus de séquençage au laboratoire. (Sélectionnez tout ce qui s'applique)

Fluorimètre Qubit

Nanodrop

Lecteur de plaques fluorescentes

Analyseur de fragments (par exemple, Bioanalyzer ou TapeStation)

Aucun

Autre (à préciser)

1. Le laboratoire a-t-il accès à un ou plusieurs ordinateurs spécifiquement destinés à l'analyse bioinformatique des données de séquençage ?

Oui

Non

1. Quelle est la vitesse de téléchargement de l'internet ou les analyses bioinformatiques sont effectuées ([tester ici](https://fast.com/)) ?

Chargement inférieur à 10 mbps

Chargement supérieur à 10mbps

Téléchargement inférieur à 10mbps

Téléchargement supérieur à 10mpbs

1. Parmi les outils bioinformatiques suivants, lesquels sont utilisés ?

Espace Base

EPI2ME

MinKNOW

Outils CGE (ResFinder, VirulenceFinder, PlasmidFinder, SerotypeFinder)

Souche suivante/Nextraclade

FluServer

GISAID EPIFLU/EPICOV

Terra.bio

CLC Genomics

BioNumérique

Généreux

IGV

IRMA

MIRA

DNAStar

BioEdit

EDGE

MEGA

Galaxie

Système de serveur Ion Reporter

Système d'exploitation Ubuntu

Autre (à préciser)

Aucune de ces réponses

1. Où les données de séquençage sont-elles sauvegardée à long terme ? (Sélectionnez toutes les réponses qui s'appliquent)

Serveur de fichiers

Disque dur externe

Disque dur de l'ordinateur

Stockage en nuage

Autres

1. Existe-t-il une copie des données sauvegardées ?

Oui

Non

1. Où les données séquentielles sont-elles sauvegardées ?

Serveur de fichiers

Disque dur externe

Disque dur de l'ordinateur

Stockage en nuage

Aucun

Autres

1. Existe-t-il une base de données LIMS permettant de relier les séquences aux métadonnées du patient ?

Oui

Non
