# Supplementary material for: Next-generation sequencing and bioinformatics capacity: findings from a multi-country survey to guide the genomics costing tool 2.0
Source: Front Public Health. 2026 Jun 25;14:1838184. doi: 10.3389/fpubh.2026.1838184 (PMC13367074; doi:10.3389/fpubh.2026.1838184)
Supplement: SUPPLEMENTARY FILE 3 — Survey tool (Russian). [file Table_3.DOCX]

**Опросник по инструменту оценки стоимости геномного секвенирования (Genomics costing tool, GCT) – русский язык**

Данная информация будет использована для определения приоритетности компонентов, которые будут включены в обновленную версию [инструмента оценки стоимости геномного секвенирования (GCT)](https://www.who.int/publications/i/item/9789240090866" \t "_blank" HYPERLINK "https://www.who.int/publications/i/item/9789240090866" \t "_blank). ([Статья о GCT](https://doi.org/10.3389/fpubh.2024.1404243" \t "_blank" HYPERLINK "https://doi.org/10.3389/fpubh.2024.1404243" \t "_blank))

Примечание: Данные, собранные в ходе этого опроса, будут анонимизированы, чтобы исключить возможность отследить индивидуальные ответы участников. Затем анонимизированные данные будут обобщены, чтобы определить основные выводы и тенденции. Эти обобщенные результаты могут быть использованы в рукописи (манускрипте), которая будет подана на публикацию в рецензируемом журнале. Принимая участие в данном опросе, вы соглашаетесь на использование ваших анонимизированных данных в этих целях. Ваша конфиденциальность имеет для нас первостепенное значение, поэтому будут приняты все необходимые меры для защиты вашей информации.

1. Название лаборатории
2. Страна, в которой находится лаборатория
3. Тип лаборатории

Национальная

Субнациональная (региональная - внутри страны)

Другое (пожалуйста, укажите)

1. Электронная почта и номер телефона контактного лица лаборатории

Электронная почта

Номер телефона:

1. На какой(-ие) язык(-и) необходимо перевести GCT? (Выберите все применимые варианты):

Арабский

Китайский

Английский язык

Португальский (Европейский)

Французский

Русский

Испанский

Другое (пожалуйста, укажите)

1. Укажите источник финансирования геномного секвенирования? (Выберите все, что применимо)

Финансирование от партнеров/агентств, выделенное для чрезвычайной ситуации

Средства на ликвидацию последствий чрезвычайных ситуаций от вашего правительства

Годовой бюджет правительства

Долгосрочное (не менее 3 лет) финансирование от партнерских организаций

Долгосрочное финансирование не определено

Финансирование для исследований или проектов

Другое (пожалуйста, укажите)

1. Отвечает ли лаборатория за расчет стоимости?

Да - Лаборатория выполняет расчет стоимости

Нет - Расчет стоимости выполняется другой стороной

1. Отвечает ли лаборатория за закупки?

Да - Лаборатория осуществляет закупки

Нет - Закупки осуществляются другой стороной

1. Укажите все высокоприоритетные патогены, которые в настоящее время включены в эпиднадзор и требуют рутинного геномного секвенирования.

SARS-CoV-2

Грипп

Другие респираторные вирусы

Кишечные бактерии

Возбудители внутрибольничных инфекций (ВБИ)

ВИЧ

Лекарственная устойчивость к ВИЧ

Арбовирусы

*Микобактерии туберкулеза* (МТБ)

Лекарственная устойчивость к МТБ

Другое (пожалуйста, укажите)

1. Укажите приблизительное среднее количество образцов в год для каждого патогена

*Текстовые поля появляются в зависимости от ответов, указанных выше

1. Какова текущая годовая пропускная способность (количество образцов) для всех патогенов, которые подвергаются секвенированию?

0 - планирующие создать лабораторию для секвенирования

1-100

101-600

601-1000

1001-2000

2001-3000

4001-5000

5001+

1. Проводит ли ваша лаборатория секвенирование для неинфекционных заболеваний (например, онкология, генетические заболевания).

Нет

Да, пожалуйста, укажите

1. Какие типы образцов принимаются для секвенирования? (Выберите все, что применимо)

Человеческая ткань (например, ткань легкого)

Носоглоточные мазки

Мазки с ротоглотки

Носовые (средняя носовая раковина)

Мазки из передней части носа

Носоглоточный смыв/аспират или назальный смыв/аспират

Бронхоальвеолярный лаваж

Трахеальный аспират

Плевральная жидкость

Слюна

Мокрота

Кал

Цельная кровь

Сыворотка

Плазма

Другое (пожалуйста, укажите)

1. Какой(-ие) набор(-ы) для выделения нуклеиновых кислот используется для проведения секвенирования? (Выберите все, что применимо)

Наборы Qiagen Dneasy для крови и тканей (Qiagen Dneasy Blood & Tissue)

Наборы для ДНК Qiagen QIAamp (Qiagen QIAamp DNA)

Наборы для ДНК из тканей Qiagen EZ1/2 (Qiagen EZ1/2 DNA Tissue)

Набор для выделения ДНК Promega Wizard (Promega Wizard DNA Extraction)

Набор для выделения общей нуклеиновой кислоты MagMAX от Applied Biosystems (Applied Biosystems MagMAX total nucleic acid isolation)

Другое (пожалуйста, укажите)

1. Используются ли автоматические станции для выделения?

Да

Нет (перейдите к странице 10)

1. Какие автоматические станции используются для выделения? (Выберите все, что применимо)

Qiagen QIAsymphony

Qiagen EZ2 Connect

Qiagen EZ1 Advanced XL

Qiagen QIAcube Connect/HT

Roche MagnaPure

PerkinElmer Chemagic 360

ThermoFisher Kingfisher

Eppendorf EpMotion 5073t

Eppendorf EpMotion 5075t

Eppendorf EpMotion 5075v

Eppendorf EpMotion 5075vt

Другое (пожалуйста, укажите)

1. Используются ли системы для обработки жидкостей (дозирующие станции) для автоматизированной подготовки библиотек?

Да

Нет (перейдите к странице 12)

1. Какие системы для обработки жидкостей (дозирующие станции) имеются в лаборатории секвенирования? (Выберите все, что применимо)

Eppendorf EpMotion 5073t

Eppendorf EpMotion 5075t

Promega Maxprep

Beckman Coulter Biomek

PerkinElmer Sciclone

Система ClearLabs

Opentrons OT-2

Другое (пожалуйста, укажите)

1. Какие платформы от Illumina имеются у Вас в наличии? (Выберите все, что применимо)

iSeq

MiniSeq

MiSeq

NextSeq 500

NextSeq 550

NextSeq 1000/2000

Отсутсвуют (перейдите к странице 15)

Другое (пожалуйста, укажите)

1. Сколько единиц каждого прибора Illumina имеется у Вас в наличии?

* Появляются текстовые поля для ввода числа в зависимости от ответов выше

1. Какие наборы используются для проведения секвенирования с платформами от Illumina? (Выберите все, что применимо)

iSeq 100 i1 v2

MiniSeq RapidKit

MiniSeq Mid Output (300c)

MiniSeq High Output (75c)

MiniSeq High Output (150c)

MiniSeq High Output (300c)

MiSeq v2 Nano (300c)

MiSeq v2 Nano (500c)

MiSeq v2 Micro (300c)

MiSeq v2 (300c)

MiSeq v2 (500c)

MiSeq v3 (150c)

MiSeq v3 (600c)

NextSeq Mid (150c)

NextSeq Mid (300c)

NextSeq High (75c)

NextSeq High (300c)

NextSeq High (500c)

NextSeq P1 (100c)

NextSeq P1 (300c)

NextSeq P1 (600c)

NextSeq P2 v3 (100c)

NextSeq P2 v3 (200c)

NextSeq P2 v3 (300c)

NextSeq P3 (50c)

NextSeq P3 (100c)

NextSeq P3 (200c)

NextSeq P3 (300c)

NextSeq P4 (50c)

NextSeq P4 (100c)

NextSeq P4 (200c)

NextSeq P4 (300c)

NextSeq P4 (50c)

Другое (пожалуйста, укажите)

1. Какие наборы для подготовки библиотек используются при проведении секвенирования с платформами от Illumina? (Выберите все, что применимо)

NexteraXT

Illumina DNA Prep

Illumina COVIDSeq

Illumina Respiratory Virus Oligo Panel

Illumina Respiratory Pathogen ID/AMR Enrichment Panel Kit

Другое (пожалуйста, укажите)

1. Загружены ли постановки на платформах Illumina на полную мощность?

Да – Загрузка постановок всегда оптимизирована

Иногда – Норма загрузки постановок иногда оптимизирована, а иногда постановки недогружены

Нет - Норма загрузки постановок не оптимизирована, частота, с которой поступают образцы, не допускает оптимизации загрузки постановок

1. Используются ли платформы для секвенирования от Illumina совместно с другими лабораториями (командами) или только для вашей лаборатории?

Да – платформы используются совместно

Нет – только для нашей лаборатории

Другое (пожалуйста, укажите)

1. Какие платформы ONT имеются у Вас в наличии? (Выберите все, что применимо)

MinION Mk1C

MinION Mk1D

MinION Mk1B

GridION

PromethION (включая P2 и P2 Solo)

Нет (перейдите к странице 18)

Другое (пожалуйста, укажите)

1. Сколько единиц каждого прибора от ONT имеется у Вас в наличии?

* Появляются текстовые поля для ввода числа в зависимости от ответов выше

1. Какие наборы для подготовки библиотек используются при проведении секвенирования с платформами от ONT? (Выберите все, что применимо)

Набор лигирования для секвенирования V14 (Ligation Sequencing Kit V14, SQK-LKS114)

Набор для штрихкодирования 16S 1-24 (16S Barcoding Kit 1-24, SQK-16S023)

Набор для быстрого секвенирования V14 (Rapid Sequencing Kit V14, SQK-RAD114)

Набор для секвенирования ультрадлинной ДНК V14 (Ultra-Long DNA Sequencing Kit V14, SQK-ULK114)

Набор для быстрого ПЦР-штрихкодирования (Rapid PCR Barcoding Kit, SQK-RPB004)

Расширение Midnight RT PCR (Midnight RT PCR Expansion, EXP-MRT001)

Набор для быстрого штрихового кодирования (Rapid Barcoding Kit, SQK-RBK110.96)

Другое (пожалуйста, укажите)

1. Используется ли оборудование для секвенирования ONT совместно с другими лабораториями (командами)?

Да

Нет

Другое (пожалуйста, укажите)

1. Загружены ли постановки на платформах ONT на полную мощность?

Да – Загрузка постановок всегда оптимизирована

Иногда – Норма загрузки постановок иногда оптимизирована, а иногда постановки недогружены

Нет - Норма загрузки постановок не оптимизирована, частота, с которой поступают образцы, не допускает оптимизации загрузки постановок

1. Какие платформы для секвенирования от Thermo Fisher имеются у Вас в наличии? (Выберите все, что применимо)

Платформа Ion Chef™ (Ion Chef™ Instrument)

Система Ion OneTouch™ 2 (Ion OneTouch™ 2 System)

Платформа Ion OneTouch™ 2 (Ion OneTouch™ 2 Instrument)

План обслуживания для системы секвенирования следующего поколения Ion Torrent™ (Service Plan for Ion Torrent™ Next-Generation Sequencing System)

Система Ion GeneStudio S5 (Ion GeneStudio S5 System)

Система Ion GeneStudio S5 Plus (Ion GeneStudio S5 Plus System)

Система Ion GeneStudio S5 Prime (Ion GeneStudio S5 Prime System)

Система Ion PGM Dx (Ion PGM Dx System)

Система Ion Torrent Genexus (Ion Torrent Genexus System)

Система Genexus Purification (Genexus Purification System)

Интегрированный секвенатор Genexus (Genexus Integrated Sequencer)

Нет (перейдите к странице 21)

Другое (пожалуйста, укажите)

1. Сколько единиц каждого прибора Thermo Fisher имеется у Вас в наличии?

* Появляются текстовые поля для ввода числа в зависимости от ответов выше...

1. Какие наборы для проведения секвенирования используются с платформами от Thermo Fisher?

Набор Ion PGM™ Template OT2 400

Набор Ion PI™ IC 200

Набор Ion PI™ Template OT2 200 Kit v2

Набор Ion PI™ Template OT2 200 Kit v3

Калибровочный стандарт Ion S5™ (Ion S5™ Calibration Standard)

Набор для секвенирования Ion PGM™ 200 (Ion PGM™ 200 Sequencing Kit)

Набор Ion PGM™ Sequencing 400

Набор Ion PI™ Sequencing 200 Kit v2

Набор Ion PI™ Sequencing 200 Kit v3

Другое (пожалуйста, укажите)

1. Какие наборы для подготовки библиотек используются при проведении секвенирования с платформами от Thermo Fisher?

Набор библиотек Ion AmpliSeq™ 2.0 (Ion AmpliSeq™ Library Kit 2.0)

Набор для создания библиотеки РНК Ion AmpliSeq™ (Ion AmpliSeq™ RNA Library Kit)

Ion TargetSeq™ Custom Enrichment Kit, 100-500 kb

Ion TargetSeq™ Custom Enrichment Kit, 500 kb - 2 Mb

Ion TargetSeq™ Custom Enrichment Kit, 2-10 Mb

Набор библиотек фрагментов Ion Xpress™ Plus (Ion Xpress™ Plus Fragment Library Kit)

Набор библиотек фрагментов Ion Plus (Ion Plus Fragment Library Kit)

Набор для подготовки библиотек Thermo Scientific® MuSeek™ для платформы Ion Torrent™ (Thermo Scientific® MuSeek™ Library Preparation Kit for the Ion Torrent™ instrument)

NEBNext® Fast DNA Fragmentation & Library Prep Set for Ion Torrent

Набор для быстрой подготовки библиотек ДНК NEBNext® для Ion Torrent 4 (NEBNext® Fast DNA Library Prep Set for Ion Torrent 4)

Набор библиотек фрагментов Ion Xpress™ Plus для системы AB Library Builder™ (Ion Xpress™ Plus Fragment Library Kit for AB Library Builder™ System)

Набор библиотек фрагментов Ion Plus для системы AB Library Builder™ (Ion Plus Fragment Library Kit for AB Library Builder™ System)

Набор для метагеномики Ion 16S™ (Ion 16S™ Metagenomics Kit)

Набор библиотек Ion TrueMate™ (Ion TrueMate™ Library Kit)

Набор библиотек Ion TrueMate™ Plus (Ion TrueMate™ Plus Library Kit)

Ion Total RNA-Seq Kit v2

Модуль для очистки на магнитных частицах (Magnetic Bead Purification Module)

Адаптеры для библиотек фрагментов Ion Plus (Ion Plus Fragment Library Adapters)

Набор Ion Xpress™ RNA-Seq Barcode 1-16 (Ion Xpress™ RNA-Seq Barcode 1-16 Kit)

Комплект адаптеров для штрих-кодов Ion Xpress™ 1-16 (Ion Xpress™ Barcode Adapters 1-16 Kit)

Комплект адаптеров для штрих-кодов Ion Xpress™ 17-32 (Ion Xpress™ Barcode Adapters 17-32 Kit)

Комплект адаптеров для штрих-кодов Ion Xpress™ 33-48 (Ion Xpress™ Barcode Adapters 33-48 Kit)

Комплект адаптеров для штрих-кодов Ion Xpress™ 49-64 (Ion Xpress™ Barcode Adapters 49-64 Kit)

Комплект адаптеров для штрих-кодов Ion Xpress™ 65-80 (Ion Xpress™ Barcode Adapters 65-80 Kit)

Комплект адаптеров для штрих-кодов Ion Xpress™ 81-96 (Ion Xpress™ Barcode Adapters 81-96 Kit)

Комплект адаптеров для штрих-кодов Ion Xpress™ 1-96 (Ion Xpress™ Barcode Adapters 1-96 Kit)

Другое (пожалуйста, укажите)

1. Загружены ли постановки на платформах Thermo Fisher на полную мощность?

Да – Загрузка постановок всегда оптимизирована

Иногда – Норма загрузки постановок иногда оптимизирована, а иногда постановки недогружены

Нет - Норма загрузки постановок не оптимизирована, частота, с которой поступают образцы, не допускает оптимизации загрузки постановок

1. Используются ли оборудование для секвенирования от Thermo Fisher совместно с другими лабораториями (командами) или только для вашей лаборатории?

Да – платформы используются совместно

Нет – только для нашей лаборатории

Другое (пожалуйста, укажите)

1. Какие платформы для секвенирования от MGI имеются у Вас в наличии? (Выберите все, что применимо)

DNBSEQ-T7

DNBSEQ-G400

DNBSEQ-G50

DNBSEQ-G99

DNBSEQ-E25

Нет (перейдите к странице 24)

Другое (пожалуйста, укажите)

1. Сколько единиц каждого прибора MGI имеется у Вас в наличии?

* Появляются текстовые поля для ввода числа в зависимости от ответов выше

1. Какие наборы для подготовки библиотек используются при проведении секвенирования с платформами от MGI? (Выберите все, что применимо)

Набор для быстрой подготовки библиотек без ПЦР MGIEasy FS V2.0 (MGIEasy Fast PCR-FREE FS Library Prep Set V2.0)

Набор для быстрой подготовки библиотек MGIEasy FS V2.0 (MGIEasy Fast FS Library Prep Set V2.0)

Универсальный набор для подготовки библиотек MGIEasy Duplex UMI (MGIEasy Duplex UMI Universal Library Prep Set)

Универсальный набор для подготовки библиотек MGIEasy UDB (MGIEasy UDB Universal Library Prep Set)

Набор для быстрой подготовки библиотек РНК MGIEasy (MGIEasy Fast RNA Library Prep Set)

Набор для подготовки библиотек ДНК без ПЦР MGIEasy (MGIEasy PCR-Free DNA Library Prep Set)

Набор для подготовки библиотек ДНК без ПЦР MGIEasy FS (MGIEasy FS PCR-Free DNA Library Prep Set)

Набор для подготовки библиотеки РНК MGIEasy (MGIEasy RNA Library Prep Set)

Набор для подготовки библиотек ДНК MGIEasy FS (MGIEasy FS DNA Library Prep Set)

Универсальный набор для подготовки библиотек ДНК MGIEasy (MGIEasy Universal DNA Library Prep Set)

Набор для быстрой подготовки библиотек ДНК MGIEasy FS (MGIEasy Fast FS DNA Library Prep Set)

Набор для подготовки библиотек геномов респираторных микроорганизмов MGIEasy (MGIEasy Respiratory Microorganism Genome Library Preparation Set)

Другое (пожалуйста, укажите)

1. Какие наборы для проведения секвенирования используются с платформами от MGI? (Выберите все, что применимо)

Набор для секвенирования с высокой пропускной способностью DNBSEQ-G400 (DNBSEQ-G400 High-throughput Sequencing Set)

DNBSEQ-G400 Набор для быстрого секвенирования с высокой пропускной способностью (DNBSEQ-G400 High-throughput Rapid Sequencing Set)

Набор для (быстрого) секвенирования с высокой пропускной способностью DNBSEQ-G50RS (DNBSEQ-G50RS High-throughput (Rapid) Sequencing Set)

Набор для секвенирования с высокой пропускной способностью DNBSEQ G99 (DNBSEQ G99 High-throughput Sequencing Set)

Набор для секвенирования с высокой пропускной способностью DNBSEQ-T7RS (DNBSEQ-T7RS High-throughput Sequencing Set)

Другое (пожалуйста, укажите)

1. Загружены ли постановки на платформах MGI на полную мощность?

Да – Загрузка постановок всегда оптимизирована

Иногда – Норма загрузки постановок иногда оптимизирована, а иногда постановки недогружены

Нет - Норма загрузки постановок не оптимизирована, частота, с которой поступают образцы, не допускает оптимизации загрузки постановок

1. Используются ли оборудование для секвенирования от MGI совместно с другими лабораториями (командами) или они предназначены только для вашей лаборатории?

Да – платформы используются совместно

Нет – только для нашей лаборатории

Другое (пожалуйста, укажите)

1. Пожалуйста, выберите любое другое оборудование для секвенирования, имеющееся в лаборатории (выберите все, что применимо)

Секвенирование по методу Сэнгера - генетический анализатор ABI (ABI Genetic Analyzer)

Секвенирование по методу Сэнгера - Promega Spectrum

Ultima Genomics

Element Biosciences

Pacific Bio (PacBio)

Другое оборудование для секвенирования отсутствует

Другое (пожалуйста, укажите)

1. Пожалуйста, выберите приборы, доступные для контроля качества процесса секвенирования в лаборатории. (Выберите все, что применимо)

Флуорометр Qubit

Нанодроп

Флуоресцентный планшетный ридер

Анализатор фрагментов (например, Bioanalyzer или TapeStation)

Нет в наличии

Другое (пожалуйста, укажите)

1. Есть ли в лаборатории доступ к компьютеру (компьютерам), специально предназначенному(-ым) для биоинформационного анализа данных секвенирования?

Да

Нет

1. Какова скорость загрузки/выгрузки (download/upload) интернета? ([Вы можете проверить скорость здесь](https://fast.com/))

Выгрузка (upload) менее 10 мбит/с

Выгрузка (upload) более 10 мбит/с

Загрузка (download) менее 10 мбит/с

Загрузка (download) более 10 мбит/с

1. Какие из перечисленных ниже биоинформационных инструментов вы используете?

BaseSpace

EPI2ME

MinKNOW

Инструменты CGE (ResFinder, VirulenceFinder, PlasmidFinder, SerotypeFinder)

Nextstrain/Nextclade

FluServer

GISAID EPIFLU/EPICOV

Terra.bio

CLC Genomics

BioNumerics

Geneious

IGV

IRMA

MIRA

DNAStar

BioEdit

EDGE

MEGA

Galaxy

Серверная система Ion Reporter (Ion Reporter Server System)

Операционная система Ubuntu (Ubuntu Operating System)

Другое (пожалуйста, укажите)

Ничего из вышеперечисленного

1. Где хранятся данные секвенирования в долгосрочной перспективе? (Выберите все, что применимо)

Файловый сервер

Внешний жесткий диск

Жесткий диск компьютера

Облачное хранилище

Другое

1. Существует ли резервное копирование данных секвенирования?

Да

Нет

1. Где хранятся резервные копии данных секвенирования?

Файловый сервер

Внешний жесткий диск

Жесткий диск компьютера

Облачное хранилище

Нет

Другие

1. Существует ли база данных ЛИС для связи последовательностей с метаданными?

Да

Нет
