# Supplementary material for: Next-generation sequencing and bioinformatics capacity: findings from a multi-country survey to guide the genomics costing tool 2.0
Source: Front Public Health. 2026 Jun 25;14:1838184. doi: 10.3389/fpubh.2026.1838184 (PMC13367074; doi:10.3389/fpubh.2026.1838184)
Supplement: SUPPLEMENTARY FILE 4 — Survey tool (Spanish). [file Table_4.DOCX]

Encuesta de la herramienta de cálculo de costes genómicos - Espanol

Esta información se utilizará para apoyar la priorización de componentes que se incluirán en una versión actualizada de la [Herramienta de cálculo de costes genómicos](https://www.who.int/publications/i/item/9789240090866). Artículo GCT:

Nota: Los datos recoleccionados en esta encuesta se anonimizarán para garantizar que las respuestas individuales no puedan atribuirse a ningún participante. A continuación, los datos anónimos se resumirán para destacar las principales conclusiones y tendencias. Estos resultados resumidos podrán utilizarse en un manuscrito que se presentará para su publicación en una revista revisada por expertos. Al participar en esta encuesta, da su consentimiento para que se utilicen sus datos anónimos con estos fines. Su privacidad y confidencialidad son de suma importancia para nosotros, y se tomarán todas las medidas necesarias para proteger su información.

.

1. Nombre del laboratorio
2. País del Laboratorio
3. Tipo de laboratorio

Nacional

Subnacional

Otros (especifique)

1. Correo electrónico y número de teléfono del punto de contacto del laboratorio:

Correo electrónic

número de teléfono

1. ¿Qué idioma(s) sería(n) más beneficioso(s) para traducir las GCT? (Seleccione todas las que opciones que apliquen ):

Árabe

Chino

Inglés

Portugués europeo

Francés

Ruso

Español

Otros (especifique por favor)

1. ¿Cuál es la fuente de financiación de la secuenciación genómica? (Seleccione todas las que opciones que apliquen ):

Financiación de emergencia de socios/agencias

Fondos de respuesta a emergencias de su Gobierno

Presupuesto anual del Gobierno

Financiación a largo plazo (al menos 3 años) de organizaciones asociadas

No se ha identificado financiación a largo plazo

Financiación de la investigación o de proyectos

Otros (especifique)

1. ¿Es el laboratorio responsable del cálculo de costes?

Sí - El laboratorio completa el cálculo de costes

No - El cálculo de costes se realiza externamente

1. ¿Es el laboratorio responsable de las adquisiciones?

Sí - El laboratorio completa la adquisición

No - La contratación se realiza externamente

1. Identificar todos los patógenos de alta prioridad actualmente bajo vigilancia que requieren secuenciación genómica rutinaria.

SARS-CoV-2

Gripe

Otros virus respiratorios

Bacterias entéricas

Infección adquirida en el hospital

VIH

Resistencia a los medicamentos del VIH

Arbovirus

micobacteria tuberculosis

Resistencia a los medicamentos micobacteria tuberculosis

Otros (especifique)

1. Introduzca una media estimada del rendimiento anual (número de muestras) para cada patógeno
2. ¿Cuál es el actual rendimiento anual de todos los patogenos secuenciados?

0 - busca establecer un laboratorio de secuenciación

1-100

101-600

601-1000

1001-2000

2001-3000

3001-4000

4001+

1. ¿Realiza su laboratorio secuenciación para modalidades de enfermedades no infecciosas (por ejemplo, oncología, genética humana)?

No

Sí, explique

1. ¿Qué tipos de muestras se aceptan para la secuenciación? (Seleccione todas las que opciones que apliquen):

Tejido humano (por ejemplo, tejido pulmonar)

Hisopos nasofaríngeos (PN)

Hisopos orofaríngeos (OP)

Turbina nasal media (NMT)

Hisopos nasales anteriores

Lavado/aspirado nasofaríngeo o lavado/aspirado nasal

Lavado broncoalveolar

Aspirado traqueal

Líquido pleural

Saliva

Esputo

Taburete

Sangre total

Suero

Plasma

Otros (especifique)

1. ¿Qué kit(s) de extracción se utilizan para la extracción de ácido nucleico para secuenciación? (Seleccione todas las que opciones que apliquen ):

Qiagen Dneasy Blood & Tissue Kits

Kits de ADN Qiagen QIAamp

Qiagen EZ1/2 DNA Tissue Kit

Kit de extracción de ADN Promega Wizard

Kit de aislamiento de ácido nucleico total MagMAX de Applied Biosystems

Otros (especifique)

1. ¿Se utilizan plataformas automatizadas de extracción?

Sí

No

1. ¿Qué plataformas de extracción automatizada se utilizan? (Seleccione todas las que opciones que apliquen ):

Qiagen QIAsymphony

Qiagen EZ2 Connect

Qiagen EZ1 Advanced XL

Qiagen QIAcube Connect/HT

Roche MagnaPure

PerkinElmer Chemagic 360

ThermoFisher Kingfisher

Eppendorf EpMotion 5073t

Eppendorf EpMotion 5075t

Eppendorf EpMotion 5075v

Eppendorf EpMotion 5075vt

Otros (especifique)

1. ¿Se utiliza algún sistema de manipulación de líquidos para la preparación automatizada de bibliotecas?

Sí

No

1. ¿De qué (si los hay) dispositivos de manipulación de líquidos dispone el laboratorio de secuenciación? (Seleccione todas las que opciones que apliquen ):

Eppendorf EpMotion 5073t

Eppendorf EpMotion 5075t

Promega Maxprep

Beckman Coulter Biomek

PerkinElmer Sciclone

Sistema ClearLabs

Opentrons OT-2

Otros (especifique)

1. ¿Qué instrumentos de Illumina tienen disponibles? (Seleccione todos los que corresponda)

iSeq

MiniSeq

MiSeq

NextSeq 500

NextSeq 550

NextSeq 1000/2000

Ninguno

Otros (especifique)

1. ¿Cuántos instrumentos Illumina de cada tipo hay disponibles?
2. ¿Qué kits de secuenciación de Illumina se utilizan? (Seleccione todas las que opciones que apliquen ):

iSeq 100 i1 v2

MiniSeq RapidKit

MiniSeq Salida Media (300c)

MiniSeq High Output (75c)

MiniSeq High Output (150c)

MiniSeq de alto rendimiento (300c)

MiSeq v2 Nano (300c)

MiSeq v2 Nano (500c)

MiSeq v2 Micro (300c)

MiSeq v2 (300c)

MiSeq v2 (500c)

MiSeq v3 (150c)

MiSeq v3 (600c)

NextSeq Mid (150c)

NextSeq Mid (300c)

NextSeq Alto (75c)

NextSeq Alto (300c)

NextSeq Alto (500c)

NextSeq P1 (100c)

NextSeq P1 (300c)

NextSeq P1 (600c)

NextSeq P2 v3 (100c)

NextSeq P2 v3 (200c)

NextSeq P2 v3 (300c)

NextSeq P3 (50c)

NextSeq P3 (100c)

NextSeq P3 (200c)

NextSeq P3 (300c)

NextSeq P4 (50c)

NextSeq P4 (100c)

NextSeq P4 (200c)

NextSeq P4 (300c)

NextSeq P4 (50c)

Otros (especifique)

1. ¿Qué kits de preparación de bibliotecas se utilizan para la secuenciación Illumina? (Seleccione todas las que opciones que apliquen ):

NexteraXT

Preparación de ADN Illumina

Illumina COVIDSeq

Panel Illumina Respiratory Virus Oligo

Kit de enriquecimiento Illumina Respiratory Pathogen ID/AMR Enrichment Panel

Otros (especifique)

1. ¿Se cargan las tiradas de Illumina al máximo de su capacidad?

Sí - Capacidad de carga siempre optimizada

A veces - La capacidad de carga está a veces optimizada y a veces infracargada

No - La capacidad de carga no está optimizada, la frecuencia del volumen de muestra es demasiado baja para la optimización de la capacidad de carga

1. ¿Los instrumentos de secuenciación de Illumina se comparten con otros grupos de laboratorio o son exclusivos de su grupo?

Sí - compartido

No - dedicado

Otros (especifique)

1. ¿De qué instrumentación ONT dispone el laboratorio de secuenciación? (Seleccione todos los que correspondan)

MinION Mk1C

MinION Mk1D

MinION Mk1B

GridION

PromethION (incluidos P2 y P2 Solo)

Ninguno

Otros (especifique)

1. ¿Cuántos instrumentos ONT de cada tipo hay disponibles?
2. ¿Qué kits de preparación de bibliotecas se utilizan para la secuenciación ONT? (Seleccione todos los que correspondan)

Kit de secuenciación de ligadura V14 (SQK-LKS114)

Kit de códigos de barras 16S 1-24 (SQK-16S023)

Kit de secuenciación rápida V14 (SQK-RAD114)

Kit de secuenciación ultralarga de ADN V14 (SQK-ULK114)

Kit de codificación rápida por PCR (SQK-RPB004)

Expansión Midnight RT PCR (EXP-MRT001)

Kit de código de barras rápido (SQK-RBK110.96)

Otros (especifique)

1. ¿Se comparte entre los grupos la instrumentación de secuenciación de la ONT?

Sí

No

Otros (especifique)

1. ¿Se cargan las tiradas de ONT al máximo de su capacidad?

Sí - Capacidad de carga siempre optimizada

A veces - La capacidad de carga está a veces optimizada y a veces infracargada

No - La capacidad de carga no está optimizada, la frecuencia del volumen de muestra es demasiado baja para la optimización de la capacidad de carga

1. ¿Qué instrumentos de secuenciación de Thermo Fisher tienen disponibles? (Seleccione todos los que corresponda, por favor detalle cuántos de cada uno en la sección de comentarios)

Ion Chef™ Instrumento

Sistema Ion OneTouch™ 2

Instrumento Ion OneTouch™ 2

Plan de servicio para el sistema de secuenciación de próxima generación Ion Torrent™

Sistema Ion GeneStudio S5

Sistema Ion GeneStudio S5 Plus

Sistema Ion GeneStudio S5 Prime

Sistema Ion PGM Dx

Sistema Ion Torrent Genexus

Sistema de purificación Genexus

Secuenciador integrado Genexus

1. ¿Cuántos instrumentos Thermo Fisher de cada tipo hay disponibles?
2. ¿Qué kits de secuenciación de Thermo Fisher se utilizan?

Ion PGM™ Plantilla OT2 400 Kit

Ion PI™ IC 200 Kit

Ion PI™ Plantilla OT2 200 Kit v2

Ion PI™ Plantilla OT2 200 Kit v3

Estándar de calibración Ion S5

Ion PGM™ 200 Kit de secuenciación

Ion PGM™ Kit de secuenciación 400

Ion PI™ Secuenciación 200 Kit v2

Ion PI™ Secuenciación 200 Kit v3

Otros (especifique)

1. ¿Qué kits de preparación de bibliotecas se utilizan para la secuenciación Thermo Fisher?

Ion AmpliSeq™ Library Kit 2.0

Ion AmpliSeq™ Kit de bibliotecas de ARN

Ion TargetSeq™ Kit de enriquecimiento personalizado, 100-500 kb

Ion TargetSeq™ Kit de enriquecimiento personalizado, 500 kb-2 Mb

Kit de enriquecimiento personalizado Ion TargetSeq™, 2-10 Mb

Kit de biblioteca de fragmentos Ion Xpress™ Plus

Kit de biblioteca de fragmentos Ion Plus

Thermo Scientific® MuSeek™ Kit de preparación de bibliotecas para el instrumento Ion Torrent™.

NEBNext® Fast DNA Fragmentation & Library Prep Set para Ion Torrent

NEBNext® Fast DNA Library Prep Set para Ion Torrent 4

Kit de bibliotecas de fragmentos Ion Xpress™ Plus para el sistema AB Library Builder™.

Kit de bibliotecas de fragmentos Ion Plus para el sistema AB Library Builder

Kit de metagenómica Ion 16S

Kit de bibliotecas Ion TrueMate

Kit de bibliotecas Ion TrueMate™ Plus

Ion Total RNA-Seq Kit v2

Módulo de purificación de perlas magnéticas

Adaptadores de bibliotecas de fragmentos Ion Plus

Ion Xpress™ RNA-Seq Barcode 1-16 Kit

Ion Xpress™ Adaptadores de código de barras 1-16 Kit

Ion Xpress™ Adaptadores de código de barras 17-32 Kit

Ion Xpress™ Adaptadores de Código de Barras 33-48 Kit

Ion Xpress™ Adaptadores de Código de Barras 49-64 Kit

Ion Xpress™ Adaptadores Codigo de Barras 65-80 Kit

Ion Xpress™ Adaptadores Código de Barras 81-96 Kit

Ion Xpress™ Adaptadores de Código de Barras 1-96 Kit

1. ¿Se cargan las tiradas de Thermo Fisher al máximo de su capacidad?

Sí - Capacidad de carga siempre optimizada

A veces - La capacidad de carga está a veces optimizada y a veces infracargada

No - La capacidad de carga no está optimizada, la frecuencia del volumen de muestra es demasiado baja para la optimización de la capacidad de carga

1. ¿Los instrumentos de secuenciación de Thermo Fisher se comparten con otros grupos de laboratorio o son exclusivos de su grupo?

Sí - compartido

No - dedicado

Otros (especifique)

1. ¿De qué instrumentos de secuenciación de MGI dispone? ((Seleccione todos los que procedan))

DNBSEQ-T7

DNBSEQ-G400

DNBSEQ-G50

DNBSEQ-G99

DNBSEQ-E25

1. ¿Cuántos instrumentos MGI de cada tipo hay disponibles?
2. ¿Qué kits de preparación de bibliotecas MGI se utilizan? (Seleccione todos los que procedan)

MGIEasy Fast PCR-FREE FS Library Prep Set V2.0

MGIEasy Fast FS Library Prep Set V2.0

Juego universal de preparación de bibliotecas MGIEasy Duplex UMI

Conjunto universal de preparación de bibliotecas MGIEasy UDB

Juego de preparación rápida de bibliotecas de ARN MGIEasy

Set de preparación de bibliotecas de ADN MGIEasy sin PCR

Set de preparación de bibliotecas de ADN MGIEasy FS sin PCR

Juego de preparación de bibliotecas de ARN MGIEasy

Set de preparación de bibliotecas de ADN MGIEasy FS

Juego universal de preparación de bibliotecas de ADN MGIEasy

Set de preparación de bibliotecas de ADN MGIEasy Fast FS

MGIEasy Conjunto de preparación de bibliotecas de genomas de microorganismos respiratorios

Otros (especifique)

1. ¿Qué kits de secuenciación se utilizan para la secuenciación MGI? (Seleccione todas las que opciones que apliquen ):

Juego de secuenciación de alto rendimiento DNBSEQ-G400

Juego de secuenciación rápida de alto rendimiento DNBSEQ-G400

DNBSEQ-G50RS Juego de secuenciación de alto rendimiento (rápida)

Conjunto de secuenciación de alto rendimiento DNBSEQ G99

Juego de secuenciación de alto rendimiento DNBSEQ-T7RS

Otros (especifique)

1. ¿Se cargan las tiradas de MGI al máximo de su capacidad?

Sí - Capacidad de carga siempre optimizada

A veces - La capacidad de carga está a veces optimizada y a veces infracargada

No - La capacidad de carga no está optimizada, la frecuencia del volumen de muestra es demasiado baja para la optimización de la capacidad de carga

1. ¿Los instrumentos del MGI se comparten con otros grupos de laboratorio o son exclusivos de su grupo?

Sí - compartido

No - dedicado

Otros (especifique)

1. Por favor, seleccione cualquier otro instrumento de secuenciación disponible en el laboratorio (Seleccione todo lo que corresponda)

Secuenciación Sanger - Analizador genético ABI

Secuenciación Sanger - Promega Spectrum

Ultima Genomics

Biociencias Elementales

Pacific Bio (PacBio)

NA

Otros (especifique)

1. Por favor, seleccione la instrumentación disponible para el control de calidad del proceso de secuenciación para el laboratorio. (Seleccione todas las opciones que correspondan)

Fluorómetro Qubit

Nanodrop

Lector de placas fluorescentes

Analizador de fragmentos (es decir, Bioanalyzer o TapeStation)

Ninguno

Otros (especifique)

1. ¿Dispone el laboratorio de uno o varios ordenadores específicos para el análisis bioinformático de los datos de secuenciación?

Sí

No

1. ¿Cuál es la velocidad de carga y descarga de Internet? ([pruebe aquí](https://fast.com/))

Carga inferior a 10 mbps

Carga superior a 10 mbps

Descarga inferior a 10mbps

Descarga a más de 10 mpbs

1. ¿Cuál de las siguientes herramientas bioinformáticas se utiliza?

BaseSpace

EPI2ME

MinKNOW

Herramientas CGE (ResFinder, VirulenceFinder, PlasmidFinder, SerotypeFinder)

Nextstrain/Nextclade

FluServer

GISAID EPIFLU/EPICOV

Terra.bio

CLC Genómica

BioNumerics

Geneious

IGV

IRMA

MIRA

DNAStar

BioEdit

EDGE

MEGA

Galaxia

Sistema de servidor Ion Reporter

Sistema operativo Ubuntu

Otros (especifique)

Ninguna de las anteriores

1. ¿Dónde se almacenan los datos de secuenciación a largo plazo? (Seleccione todo lo que corresponda)

Servidor de archivos

Disco duro externo

Disco duro de ordenador

Almacenamiento en la nube

Otros

1. ¿Existe una copia de seguridad del almacenamiento de datos de secuenciación?

Sí

No

1. ¿Dónde se almazena la copia de seguridad de los datos secuenciales?

Servidor de archivos

Disco duro externo

Disco duro de ordenador

Ninguno

Almacenamiento en la nube

1. ¿Existe una base de datos LIMS para vincular secuencias a metadatos?

Sí

No
