# Supplementary material for: Next-generation sequencing and bioinformatics capacity: findings from a multi-country survey to guide the genomics costing tool 2.0
Source: Front Public Health. 2026 Jun 25;14:1838184. doi: 10.3389/fpubh.2026.1838184 (PMC13367074; doi:10.3389/fpubh.2026.1838184)
Supplement: SUPPLEMENTARY FILE 5 — Survey solicitation. [file Table_5.DOCX]

Following the successful launch of the [SARS-CoV-2-focused Genomics Costing Tool](https://www.who.int/publications/i/item/9789240090866) (GCT) in December 2023, the GCT working group is now expanding the tool’s scope to include other priority pathogens and sequencing technologies. To achieve this, we need a comprehensive understanding of the sequencing platforms and supporting reagents used globally for sequencing priority pathogens. We kindly request your participation in a brief survey regarding your genomic surveillance instrumentation and reagent usage. Your insights are invaluable in ensuring that the most relevant and up-to-date information is included in the next version of the WHO GCT.

Thank you for your time and participation in this important initiative.
