# Supplementary material for: Next-generation sequencing and bioinformatics capacity: findings from a multi-country survey to guide the genomics costing tool 2.0
Source: Front Public Health. 2026 Jun 25;14:1838184. doi: 10.3389/fpubh.2026.1838184 (PMC13367074; doi:10.3389/fpubh.2026.1838184)
Supplement: SUPPLEMENTARY FILE 7 — Funding sources for the laboratory stratified by country development level. [file Table_7.DOCX]

| **Source of funding**  (n = 113) | **Country Development Level** | | | |  |
| --- | --- | --- | --- | --- | --- |
|  | **High Income** | **Upper-Middle Income** | **Lower-Middle Income** | **Low Income** | **Total** |
| Emergency funding from partners/agencies | 5 | 17 | 14 | 3 | 39 |
| Emergency response funds from your government | 14 | 12 | 12 | 0 | 38 |
| Government annual budget | 31 | 17 | 5 | 0 | 53 |
| Research or project-based funding | 17 | 25 | 22 | 5 | 69 |
| Other | 3 | 4 | 4 | 0 | 11 |
| Partners/Agencies |  | 4 | 3 |  |  |
| Fee for service/private sector | 3 |  | 1 |  |  |
| **Total no. respondents reporting** | **38** | **36** | **34** | **5** | **113** |
| Long-term (≥3 years) funding from partner organizations | 8 | 3 | 9 | 1 | 21 |
| No long-term funding identified | 4 | 8 | 12 | 1 | 25 |
